# The heterogeneous driving forces behind carbon emissions change in 30 selective emerging economies

**DOI:** 10.1016/j.patter.2023.100760

**Published:** 2023-05-30

**Authors:** Shuping Li, Can Cui, Jing Meng, Yuan Li, Yuli Shan, Weichen Zhao, Priti Parikh, Jiawei Yao, Dabo Guan

**Affiliations:** 1 Institute of Blue and Green Development, Weihai Institute of Interdisciplinary Research, Shandong University, Weihai 264209, China; 2 Department of Earth System Sciences, Tsinghua University, Beijing 100080, China; 3 The Bartlett School of Sustainable Construction, University College London, London WC1E 7HB, UK; 4 School of Geography, Earth and Environmental Sciences, University of Birmingham, Birmingham B15 2TT, UK; 5 College of Architecture and Urban Planning, Tongji University, Shanghai 200092, China

**Keywords:** CO_2_ emissions, emerging economies, low-carbon pathways

## Abstract

Emerging economies are predicted to be future emission hotspots due to expected levels of urbanization and industrialization, and their CO_2_ emissions are receiving more scrutiny. However, the driving forces underlying dynamic change in emissions are poorly understood, despite their crucial role in developing targeted mitigating pathways. We firstly compile energy-related emissions of 30 selective emerging economies from 2010 to 2018. Then, three growth patterns of emissions in these economies have been identified through emission data, which imply different low-carbon pathways. Most emerging economies saw an increase of varying degrees in emissions, driven by economic growth and partly offset by better energy efficiency and improvements in energy mixes. Furthermore, the industrial structure was another factor that slowed emissions, especially in Latin America and the Caribbean. Our research contributes to the heterogeneous exploration of CO_2_ emissions produced by energy among sectors and the creation of low-carbon development pathways in emerging economies.

## Highlights

• CO_2_ emissions and economic growth in 30 selective emerging economies are estimated
• Energy intensity impels emissions to decrease, showing a large reduction potential
• Changes in the energy mix and industrial structure affect emissions decline
• The heterogeneity of energy use and sectoral emissions has emerged in economies

## The bigger picture

Increasing attention has been paid to CO_2_ emissions, which are the main source of greenhouse gas emissions (GHGs) and are closely related to temperature rise in climate change. Growing CO_2_ emissions and ambiguous low-carbon transition paths have gradually become the bright spot in emerging economies. However, due to insufficient data, our understanding of which factors are the most effective drivers of emission reduction in emerging economies remains limited. Accordingly, we try to evaluate the disparity of emission trajectories in 30 selective emerging economies according to compiled emission accounts and find the heterogeneity of sector emissions and key drivers of changes in emissions to provide some thoughts for future emission reduction policies of emerging economies.

## Graphical abstract

**Figure:**
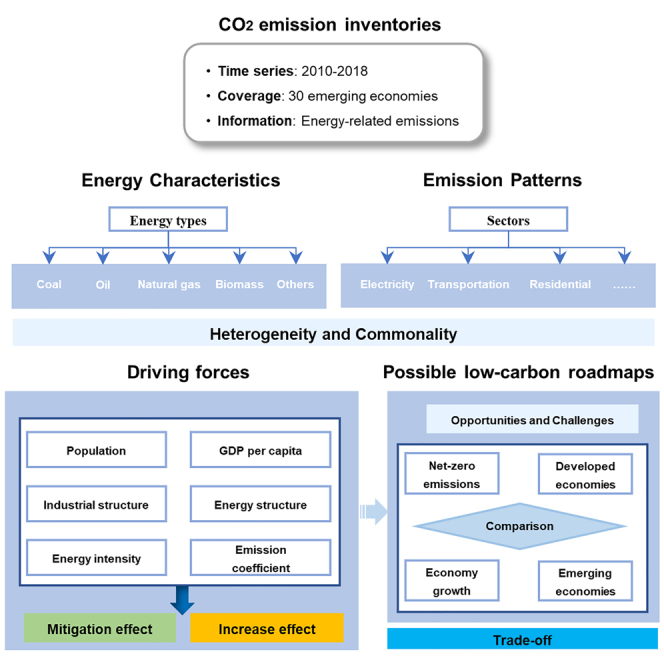

## Teaser

Emerging economies are crucial to mitigating global climate change and reducing CO_2_ emissions in the future. Through the comprehensive analysis of emission patterns and the identification of potential reduction factors in emerging economies, we can provide enhanced support for the deployment and execution of their policies.

## Introduction

To slow the progress of global climate change, nations are increasingly setting carbon reduction targets toward carbon neutrality. Most developed countries reached their peak emissions point before 2020^1^ and are pursuing energy transitions involving renewables and decarbonization.^2^^,^^3^ In contrast, the rapid industrialization and urbanization rolling out in emerging economies mean that emissions have been increasing rapidly in recent years, which presents a challenge to global climate change mitigation.^4^^,^^5^^,^^6^^,^^7^ While the per-capita emissions in emerging countries are much lower than the level associated with the 1.5°C (i.e., the Paris Agreement identifies efforts to control global temperature rise to no more than 1.5°C above pre-industrial levels by 2100) goal,^8^ it is likely that per-capita emissions in emerging economies will further rise in tandem with economic growth.^9^^,^^10^^,^^11^ The challenge facing emerging economies lies in achieving net-zero targets while simultaneously meeting the socioeconomic needs of their citizens. Therefore, trends and patterns in CO_2_ emissions and related driving factors in developing countries need investigation to add detail and nuance to strategies for low-carbon transitions.

A series of studies have analyzed emission patterns and driving forces in emerging economies, although at an aggregated regional level.^12^^,^^13^ The aggregated national CO_2_ emissions to the region level mask the heterogeneity in emissions across countries. Some recent studies have focused on individual emerging countries such as China,^14^^,^^15^ India,^16^^,^^17^^,^^18^ Malaysia,^19^ Turkey,^20^ and Ethiopia.^21^ However, it is difficult to compare the changes in emission levels and driving forces across countries due to the different data sources and time periods across studies. Recently, a few studies analyzed drivers of CO_2_ emissions in a group of developing countries. Steckel et al., by studying 20 sub-Saharan African nations, found the drivers to include population, per-capita gross domestic product (GDP), energy intensity, and carbon intensity of CO_2_ emissions and highlighted the role of rising carbon intensity in increasing emissions.^22^ Similarly, Ayompe et al., by analyzing emissions from the combustion of fossil fuels in 27 African countries, found that population and GDP were driving the growth of emissions.^9^ However, these studies focused on single regions of emerging economies and did not reveal the impact of economic structure related to emission patterns and trends, making CO_2_ emissions across regions with heterogeneous economic structures difficult to compare. More importantly, the pressure to reduce emissions varies among sectors, as do their reduction pathways. Owing to the need for socioeconomic development and coping with climate change, the transition from coal to renewable energy is, for example, deployed in the power sector,^23^^,^^24^ but the transition from fossil fuel or traditional biomass to clean fuel,^25^ as well as factors affecting CO_2_ emissions such as building area and income,^26^^,^^27^^,^^28^ are seen as taking place in the residential sector. It is thus necessary to find the key high-emission industries as well as energy with high carbon content in emerging economies before targeted emission reduction plans are formulated. Moreover, given the significant disparities in levels of economic and technological development among emerging economies, there is significant spatiotemporal heterogeneity in their energy structures, energy efficiencies, industrial structures, and CO_2_ emissions,^10^^,^^29^ which need to be analyzed one by one.

This study therefore first quantifies the driving factors of various CO_2_ emission trajectories in 30 selective emerging economies over the period from 2010 to 2018. On the one hand, fossil energy-related CO_2_ emissions in the 30 countries accounted for about 19.8% of the world’s total emissions, 1.4 times that of the United States and over 60% of China’s emissions in 2018, according to the International Energy Agency (IEA).^30^ On the other hand, the population growth rate in these emerging economies is much faster than in developed countries such as the United States. According to the World Bank,^31^ 24 of the 30 countries have population growth rates well above the 0.5% and 0.6% rates of the United States and United Kingdom, with Uganda, Tanzania, and Ethiopia experiencing population growth of up to 3.4%, 3.2%, and 2.7%, respectively. Rapid population growth signifies that these countries face higher energy demand than developed countries such as the United States and the United Kingdom, which poses challenges and risks for these countries to work toward energy transformation and emission reduction. Thus, emission patterns and potential emission reduction factors in these countries need to be mapped as soon as possible. We have used our newly built CO_2_ emission inventories, which enable transparent and comparable analysis. Thereafter, this study discusses the possible low-carbon roadmaps for these countries. This study distinguishes the heterogeneity of emissions and provides quantitative evidence for policy formulation to mitigate climate change in emerging economies.

## Results

### Economic development and national CO_2_ emission trends

**Figure 1:**
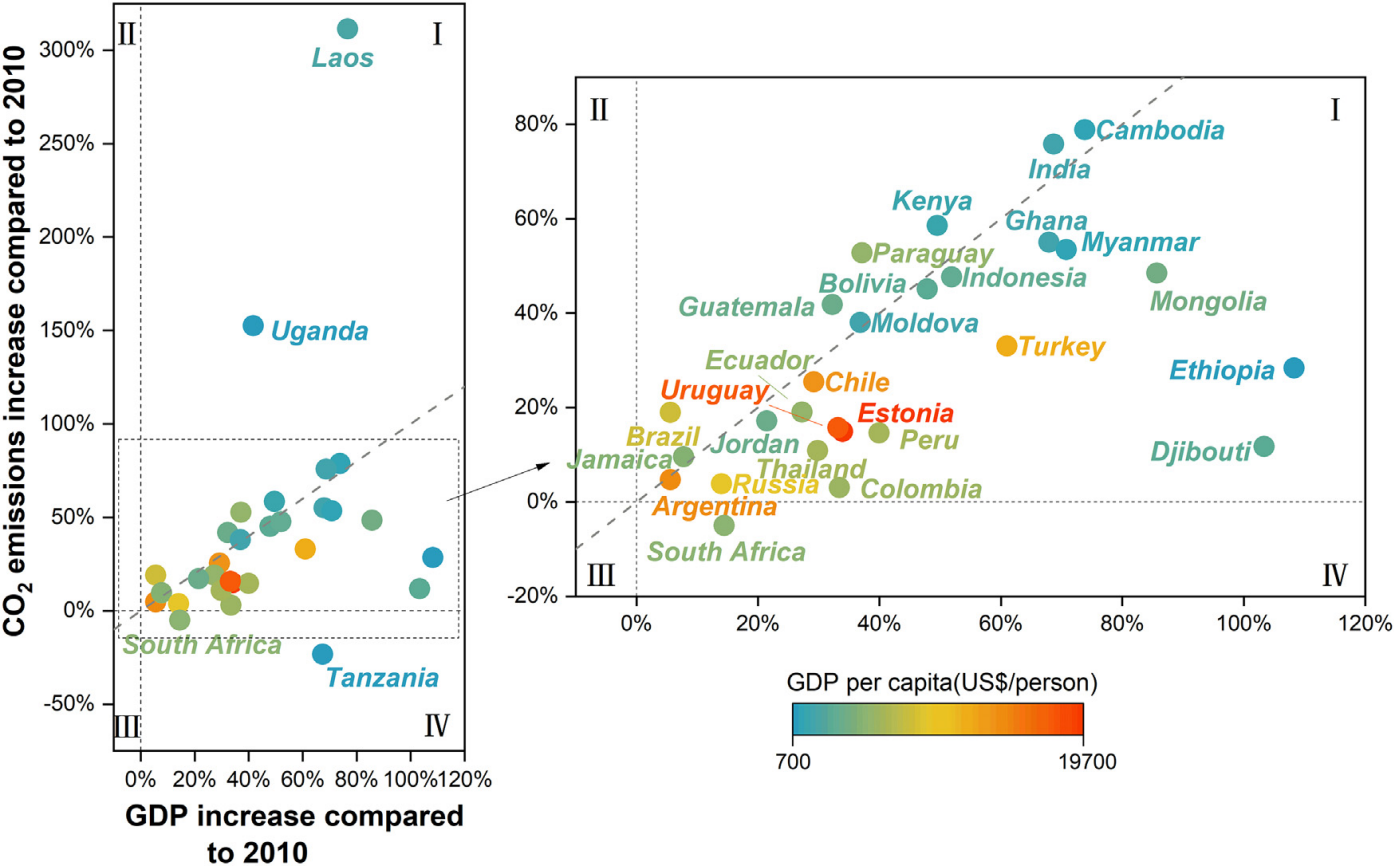
Overview of CO_2_ emissions and economic development The horizontal axis shows how much GDP in 2018 has increased compared with 2010, and the vertical axis shows how much CO_2_ emissions in 2018 have increased compared with 2010. The dashed gray line signifies the point where the rate of change of emissions is equal to the rate of change of GDP. The colored circles represent the GDP per capita for each country in 2018.

The relative growth of CO_2_ emissions and GDP (in 2018 compared with 2010) in the 30 emerging economies can be divided into three groups, as demonstrated in quadrant IV and two areas divided by a dashed gray line in quadrant I (Figure 1). South Africa and Tanzania (group one) have shown a downward trend in CO_2_ emissions and an upward trend in GDP (quadrant IV). In 2018, the GDP per capita values of South Africa and Tanzania were $5,636 and $1,052, respectively. In South Africa, CO_2_ emissions decreased by 5% from 2010 (391.3 Mt) to 2018 (372 Mt), while its economy grew by 14.4% compared with 2010—from $284.7 billion to $325.7 billion. In Tanzania, CO_2_ emissions decreased by 23.2% from 21.4 (2010) to 16.4 Mt (2018), while its GDP has grown by 67.4% from $35.4 billion to $59.3 billion. These unusual developments, of lower emissions and rising GDP, might be an exception, or South Africa and Tanzania might see emissions rebound in the future. The population of Africa is growing rapidly, which will lead to a demand for increased energy consumption for domestic, industrial, and food production needs. Meanwhile, energy access is also a challenge on the continent.^32^ About 580 million people in sub-Saharan Africa had no access to electricity in 2019,^33^ and the situation is likely to worsen under the COVID-19 pandemic. Electricity consumption and associated emissions would be highly likely to increase rapidly with the development of electricity infrastructure and an increase in energy use unless there is a rapid acceleration of investment in off-grid renewable technologies.^34^ Thus, although CO_2_ emissions in South Africa and Tanzania have declined markedly, these countries may not be able to maintain this trend in the future.

Emerging economies in the other two groups witnessed rising CO_2_ emissions and GDP. Those countries in group two have a lower growth rate of CO_2_ emissions than of GDP, which is located between the horizontal dashed line and the gray dashed line in quadrant I of Figure 1. For example, Uruguay’s rate of change in CO_2_ emissions was 15.7% in 2010–2018, which was lower than the rate of economic growth (33.2%). Similarly, the economies of Ethiopia and Djibouti developed rapidly, pushing the economic growth rate to more than 3.8 and 8.8 times that of CO_2_ emissions. The level of economic development and the GDP per capita ($19,655 in 2018) of Estonia is relatively high. The range of change in economic growth in Estonia was 33.9% in 2010–2018, and CO_2_ emissions increased by 14.9%. In recent years, the emerging economies in group three have experienced a rapid growth in CO_2_ emissions that has surpassed the GDP growth rate. Among these emerging economies, Kenya’s CO_2_ emissions in 2018 were about 1.6 times those in 2010, mainly due to rising emissions in the residential sector, where increasing use of biomass energy such as firewood for cooking has been noted. The Kenyan government introduced a zero value-added tax (VAT) for liquefied petroleum gas to reduce biomass consumption in 2016, but the subsidy was withdrawn in 2021, leading to increased reliance on biomass for cooking in low-income communities.^35^ Meanwhile, Kenya’s GDP grew by 49.5%. In addition, CO_2_ emissions have also risen significantly in Laos, Uganda, India. and Cambodia.

Carbon intensity (CO_2_ emissions per unit of GDP) is a quantitative way of emission reduction that combines the level of economic development with carbon emissions. Carbon intensity varies hugely across emerging economies; among the 30 countries studied, it ranges between 0.14 and 3.43 kg CO_2_/$ GDP. Of these, the emerging economies in Africa had relatively high carbon intensity, while the figures for those in Latin America and the Caribbean were relatively low (Figure S1). For example, in Uruguay and Argentina in 2018, the carbon intensities were approximately 0.16 and 0.25 kg CO_2_/$ GDP, respectively. This can be partly attributed to the relatively large proportion of value-added of tertiary industry in their industrial structures. By contrast, although the carbon intensity of emerging economies such as Ethiopia was decreasing year by year, it was still as high as 2.11 kg CO_2_/$ GDP in 2018. That might be down to the increasing value-added of the manufacturing and construction industry brought about by the industrialization process and the use of related emission-intensive energy and traditional biomass for cooking (Figure S1 and Table S1).

### The heterogeneity and time-series dynamic changes of energy structure

**Figure 2:**
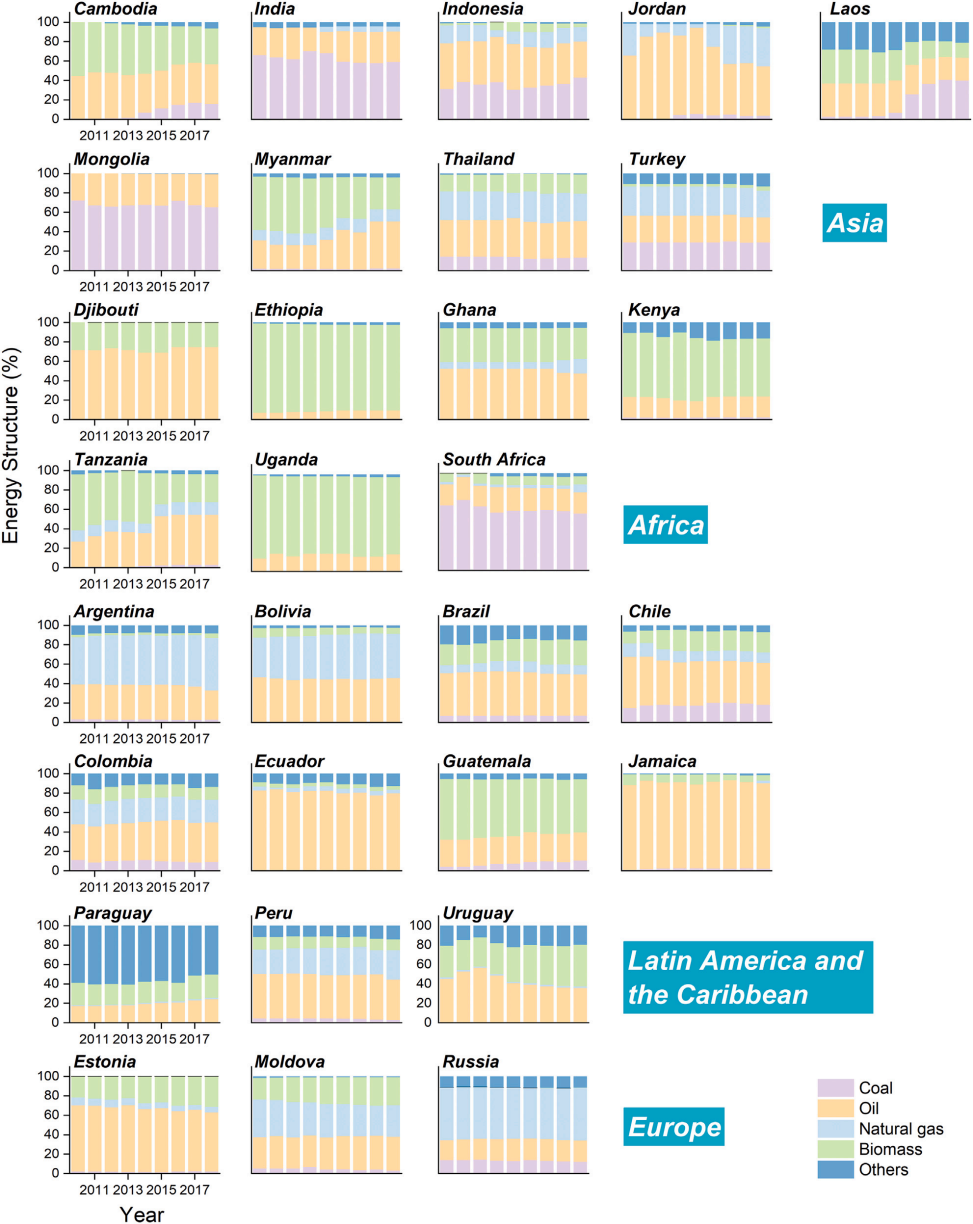
The changes of energy structure in 30 selective emerging economies, 2010–2018 The bars show the proportion in consumption about coal (purple), oil (orange), gas (light blue), biomass (green), and others (blue).

Across the emerging economies studied, there was significant variation in energy use over time. In some emerging economies of Africa and Asia, coal accounted for a relatively large proportion of the energy structure: for instance, 58.5% in South Africa and 58.9% in India in 2018 (Figure 2). Coal use in some emerging economies in these regions, including Cambodia, Laos, and Ethiopia, has increased significantly (Figure 2). In Laos, the amount of coal used in 2017 was 11.8 times that used in 2014, and the share of coal in the energy mix increased from 6.7% in 2014 to 40.6% in 2017 (Figure 2), in tandem with rapid growth in electricity generation. Likewise, the use of coal in Cambodia and Ethiopia in 2018 was some 44.2 and 13 times that in 2010, respectively. For Myanmar, though its share of oil soared from 29.1% in 2010 to 48.5% in 2017 (Figure 2), biomass still accounted for over 30% of total energy use. Similarly, the use of biomass in Kenya in 2018 was 1.5 times that used in 2010, a trend closely related to the large proportion of agricultural value added (Table S1). It is worth noting that in emerging economies such as Ethiopia, the proportion of agricultural value added exceeded that in the manufacturing and construction industry, suggesting that there is a lot of room for agricultural sector improvements in the future (Table S1). Because the majority of food production operations are now concentrated in emerging economies, decarbonizing the sector and reducing biomass use are critical to lowering emissions. In this context, the sustainable use of biomass energy in emerging economies needs to be given more consideration. Turkey’s GDP per capita in 2018, meanwhile, was quite high, topping $10,000. Its energy structure was fairly homogeneous, with coal, oil, and natural gas accounting for more than 25% of its total energy consumption.

In contrast to some emerging economies in Asia and Africa, those in Latin America and the Caribbean mainly rely on oil rather than coal as a source of energy. In Argentina, Bolivia, Peru, and Colombia, the prime energy sources are oil and gas (Figure 2); in Brazil, Chile, Guatemala, Uruguay, and Jamaica, oil and biomass energy predominate; and Ecuador depends primarily on oil and renewable energy. In terms of energy growth, the use of biomass in Argentina was more than 2-fold in 2018 compared with 2010. Bolivians increasingly turned to oil, natural gas, and renewable energy sources in addition to biomass. In Uruguay, the use of gas increased significantly (about 1.4 times in 2018 based on 2010). Meanwhile, most emerging economies with slow growth in the manufacturing and construction industry (an average annual growth rate of lower than 5% or even negative growth) and the highest proportion of service value added were located in Latin America and the Caribbean (Table S1).

### Sectoral heterogeneity of CO_2_ emissions

**Figure 3:**
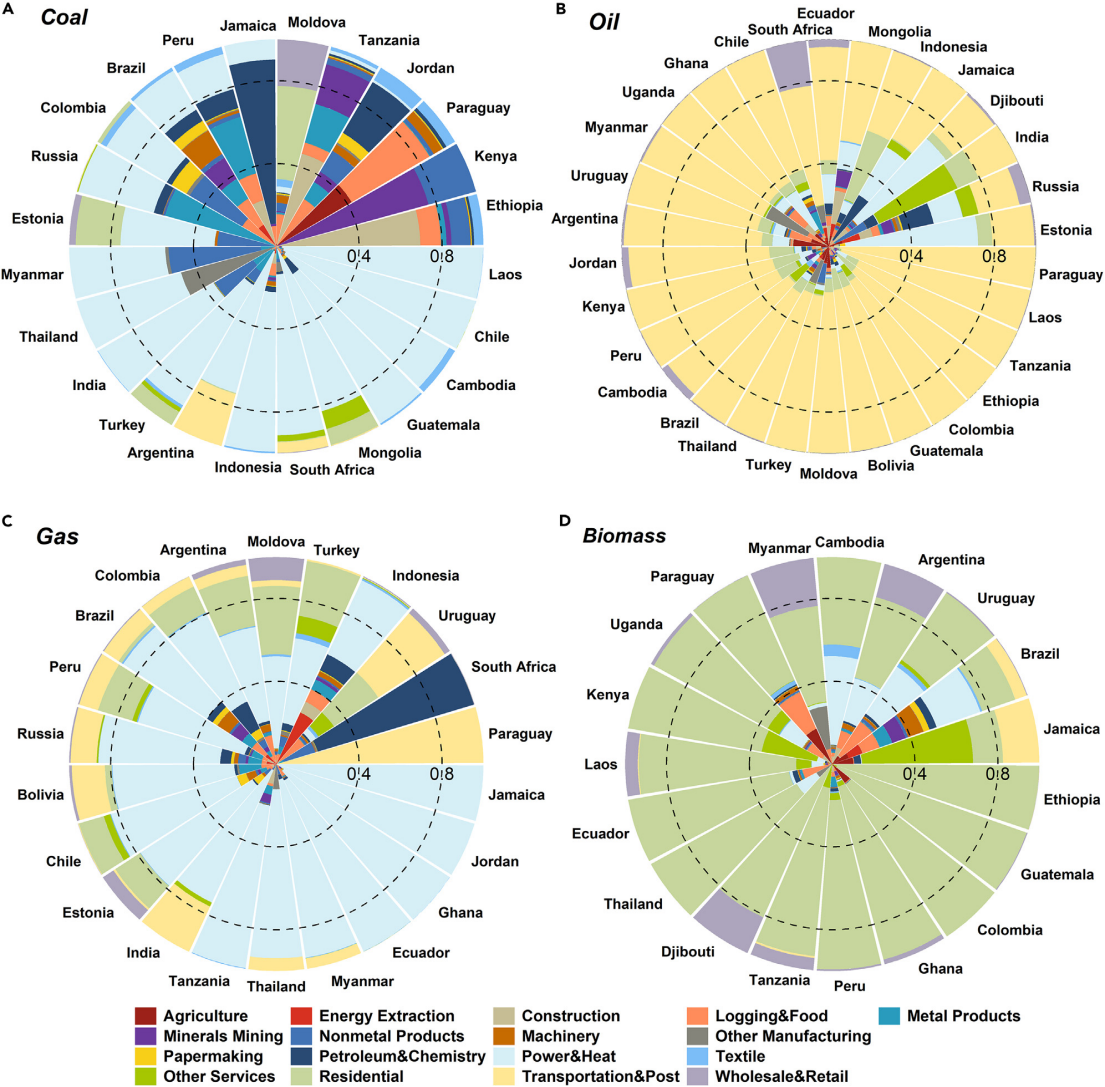
Distribution of CO_2_ emissions caused by different types of energy use among sectors in 2018 Two circles demonstrate 40% and 80% of the emissions generated by energy use. (A) The proportion of CO_2_ emissions from coal combustion is shown to be distributed among 17 sectors. (B–D) The proportion of CO_2_ emissions from oil, gas, and biomass combustion, respectively, is shown as distributed among 17 sectors.

There is significant variation in levels of emissions at the region and sector levels due to differences in the use of coal. In 2018, some emerging economies, such as India, recorded high CO_2_ emissions as a result of massive coal combustion (1,687.9 Mt) (Figure S2). In most emerging economies, the CO_2_ emissions from coal combustion were emitted primarily by the electricity and heat sector (Figure 3A). For example, in Chile and Cambodia, coal-driven CO_2_ emissions in the electricity and heating sector were as high as 96.1% and 91.5%, respectively. In Myanmar and Colombia, the nonmetal products sector was also found to be a source of coal-related emissions. CO_2_ emissions from coal combustion in Brazil, Kenya, and Moldova were more diverse in sectoral distribution. Emissions from coal in Brazil were mainly spread across the sectors of electricity and heating (21.8%), logging and food (14.6%), machinery (13.2%), energy extraction (12.1%), minerals mining (11.8%), and metal products (11%). The sectors producing most coal-driven emissions in other countries included residential sector in Moldova (45%) and minerals mining in Kenya (76.3%). It is worth mentioning in this context that most emerging economies are at the forefront of manufacturing and production processes to meet the demands of the developed world’s growing tertiary sector.

CO_2_ emissions associated with the use of oil were mainly concentrated in the transportation sector in emerging economies (yellow area in Figure 3B), while the electricity and heating sector accounted for a relatively high proportion in Estonia. Moreover, the residential sector in Indonesia accounted for 34.7% of emissions from oil use. Intuitively, there is a diversification in terms of sectoral distribution of emissions from the use of natural gas in emerging economies, including Indonesia, India, and Turkey (Figure 3C). CO_2_ emissions in Indonesia from natural gas were mainly distributed in the energy extraction and the electricity and heating sectors, while emissions in India were mainly concentrated in the transportation and the electricity and heating sectors. Natural gas-driven emissions in Turkey were mainly from the electricity and heating and residential sectors, accounting for 41.3% and 26.7%, respectively.

Emissions from the residential use of biomass in Guatemala and Colombia accounted for 96.9% and 88.3% of total biomass-driven emissions, respectively (Figure 3D). The level of traditional biomass use associated with the residential sector, which thus accounts for significant CO_2_ emissions, indicates that there is considerable room for increasing biomass efficiency in these emerging economies. Apart from those associated with residential use (mainly for cooking), emissions from biomass were also driven by sectors including electricity and heating in other emerging economies. Argentina, for instance, mainly used biomass to generate power (21.2%, or 0.6 Mt; Figures 3D and S2).

### Drivers of changes in CO_2_ emissions

**Figure 4:**
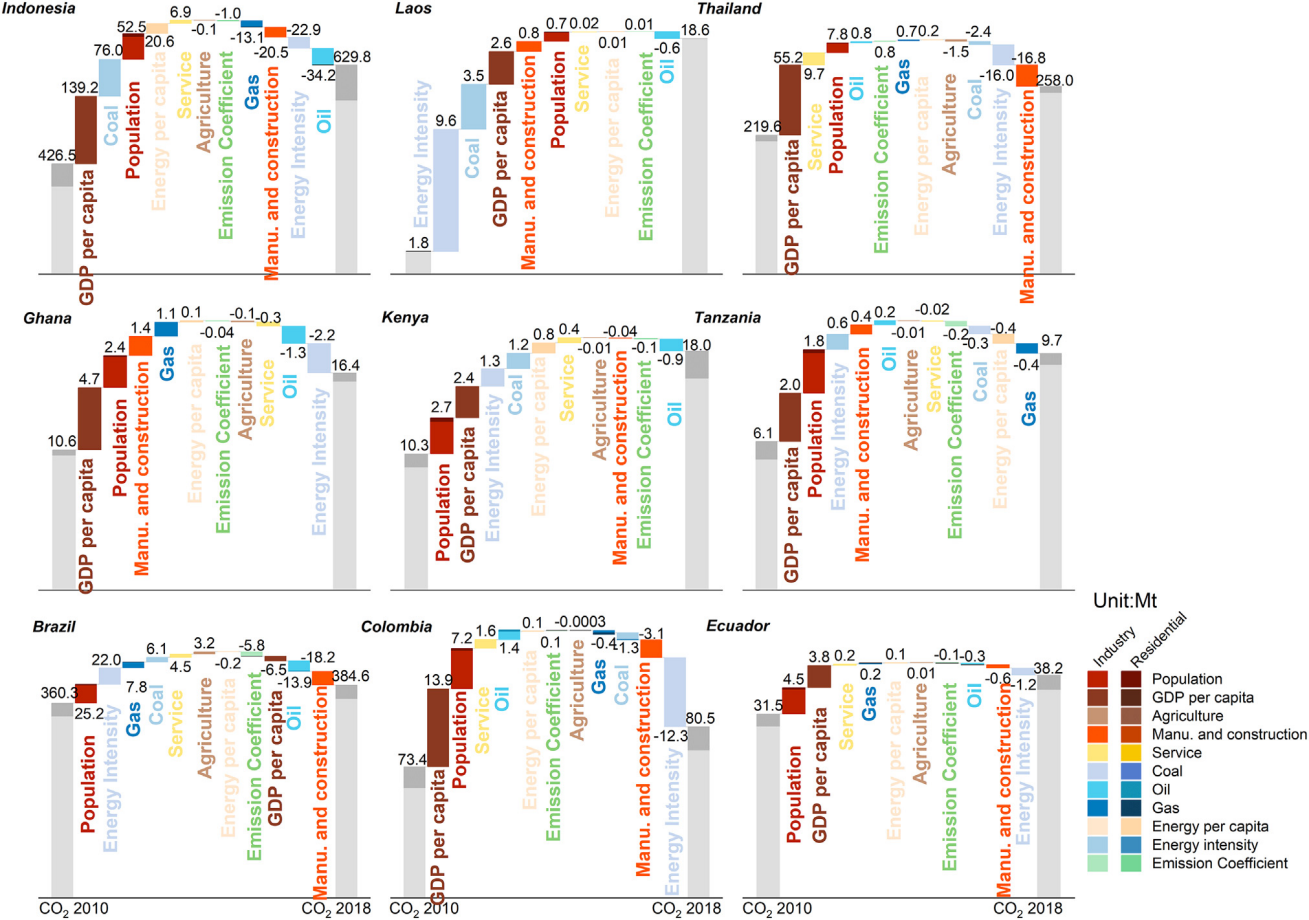
Drivers of CO_2_ emissions changes about fossil fuels, 2010–2018 The driving factors of CO_2_ emissions in the residential sector and industry are distinguished by differently colored bars and headings. Among them, the common drivers of the residential and industrial sectors are population; the share of coal, oil, and gas; and emission coefficient (CO_2_ emissions per unit energy). Energy per capita is the driving factor in the residential sector, whereas GDP per capita, energy intensity (energy use per unit GDP), and the share of the primary (agriculture), secondary (manufacturing and construction), and tertiary (service) industries are driving factors in the industrial sector.

The study analyzes the breakdown, via factors of CO_2_ emissions changes, of fossil fuels in the residential and industrial sectors (Figures 4 and S3). The drivers include emission coefficient (CO_2_ emissions per unit energy); energy structure (the share of coal, oil, and gas); population; energy intensity (energy use per unit GDP, only in the industry); industrial structure (the share of primary, secondary, and tertiary, only in the industry); GDP per capita (only in the industry); and energy per capita (only in the residential sector). With the exception of South Africa, the factors that led to a decrease in CO_2_ emissions from fossil fuels in most emerging economies from 2010 to 2018 did not ultimately counteract the causes contributing to rising CO_2_ emissions. Thus, emissions have risen across emerging economies but to varying extents. In general, a rise in GDP per capita and population were the primary drivers of CO_2_ emissions. Energy intensity, meanwhile, contributed to decreasing CO_2_ emissions in the majority of emerging economies, whereas energy structure, industrial structure, and emission coefficient had different effects across countries, driving CO_2_ emissions up or down.

The impact of energy structure on emissions differed among emerging economies. Lower coal and oil emissions were primarily responsible for this drop in emissions in Peru and Argentina, and increasing gas has contributed to a rise in CO_2_ emissions. While their lower oil use lowered overall emissions, that impact was lessened by coal use, which simultaneously raised CO_2_ emissions, particularly in Asia and including Cambodia and Laos. The case of Brazil helps in grasping how energy structure influences variations in CO_2_ emissions. Here, oil was responsible for a 13.9 Mt (3.9%) reduction in CO_2_ emissions. Coal and gas, on the other hand, have contributed to a rise in CO_2_ emissions of 6.1 (1.7%) and 7.8 Mt (2.2%), respectively. In Jordan, the most important factor in reducing CO_2_ emissions from 2010 to 2018 was reducing the share of oil in the energy mix, which resulted in a drop of 4.4 Mt (21.7%). The country’s growth in CO_2_ emissions, on the other hand, was highly dependent on its gas contribution.

With other factors remained constant, the overall industrial structure, including primary, secondary, and tertiary, in emerging economies had a variable impact on CO_2_ emissions (Figures 4 and S3). The changes in industrial structure in emerging economies were associated with a decline in such emissions, for instance in Asia (India, Indonesia, Jordan, and Thailand); Africa (South Africa); Latin America and the Caribbean (Argentina, Bolivia, Brazil, Chile, Colombia, Ecuador, Guatemala, Peru, and Uruguay); and Europe (Moldova). Different industries have had various impacts on CO_2_ emissions across emerging economies. Some emerging economies were clearly undergoing industrial structural transformation in the period studied. Manufacturing and construction triggered a drop in CO_2_ emissions, whereas the service industry had the opposite effect. For instance, in Brazil, the increase in CO_2_ emissions came from the share of service (4.5 Mt) (Figure 4). Meanwhile, manufacturing and construction contributed 18.2 Mt (5.1%) to the decrease in CO_2_ emissions in Brazil. In Thailand, service—as a pillar of the industry, especially tourism—was associated with a rise in CO_2_ emissions of 9.7 Mt (4.4%) from 2010 to 2018, while the shares of agriculture and of manufacturing and construction reduced CO_2_ emissions by 1.5 and 16.8 Mt, respectively. Yet in Mongolia, the share of manufacturing and construction boosted CO_2_ emissions by 0.6 Mt (5.4%), while the share of service triggered a reduction in CO_2_ emissions of 0.2 Mt (1.6%). When decomposition analysis includes biomass emissions, the outcomes are presented in Figure S4. Lowering biomass emissions was the main factor behind the decrease in emissions observed in Tanzania, and similar shifts in biomass emissions contributed to CO_2_ emission reduction in other economies, especially in Myanmar and Laos. Elsewhere, particularly in Kenya, biomass use has contributed to a rise in CO_2_ emissions.

## Discussion

Studies have exposed a gap between existing national policies and actions of climate change and meeting the Paris Agreement goals—that is, a bottleneck in achieving a temperature rise of no more than 2°C or 1.5°C.^36^^,^^37^ Policies to curb emissions vary across countries. The formulation of net-zero targets has created a possibility for the realization of the Paris Agreement,^38^ with 21 of the 30 selective emerging economies setting up corresponding net-zero targets (Table S1). Barring Turkey, Thailand, Indonesia, Russia, Ghana, and India, the remaining 15 of the 21 countries have set targets for carbon neutrality for 2050. Turkey and Thailand have established carbon neutrality objectives for 2053 and 2065, respectively. Ghana and India aim to achieve their net-zero emissions by 2070, with India for the first time committing to meet 50% of its energy requirements from renewable sources by 2030. Russia and Indonesia have set a 2060 target to achieve net-zero emissions. Despite the challenges, countries are now advancing their carbon neutrality targets in the form of renewable energy substitution; the usage of carbon capture, utilization, and storage (CCUS); and tapping ocean and terrestrial carbon sinks.^39^^,^^40^

Detailed emission inventories by energy types and sectors are important for the development of emission policies in emerging economies. Therefore, in order to better address climate change and global warming, we have collected energy data from 30 selective emerging economies through data crowdsourcing to complete the construction of emissions and thus analyze potential emission reduction pathways in scientific ways. In certain countries, such as South Africa and Tanzania, the relationship between economic development and CO_2_ emissions has shifted in the other direction, toward the decline of emissions and the increase of economy—although whether this is a long-term, stable trend is unknown. In the vast majority of emerging economies, economic development and emissions were still in a high-speed growth trajectory.

Numerous developed countries that are well known to the public have largely reached the emission peaks, whereas most developing countries have not and are still in the upward stage.^41^ Also, the heterogeneity of emission patterns and drivers between countries has been demonstrated in our study and previous papers.^42^^,^^43^ On the one hand, this study shows that energy intensity is the important driver for the decline of emissions in most emerging economies, especially Djibouti. By contrast, the energy intensity of some emerging economies even drives emission growth, such as in Kenya and Laos. On the other hand, the decline of energy intensity—that is, the improvement of energy efficiency—in numerous emerging economies cannot offset the increase in emissions caused by economic development and population. Thus, there is still room for optimizing energy intensity and carbon intensity to mitigate emissions.

Moreover, the adjustment of industrial structure in emerging economies has gradually become a key factor in low-carbon pathways. One of the plausible pathways for emerging economies to reduce emissions is therefore to lean toward the tertiary sectors. However, manufacturing and construction is the mainstay of emerging economies; thus, the industrial structure of these emerging economies cannot be excessively tilted to the tertiary sectors. Reduced consumption and changes in lifestyle choices will be required in developed nations to lower demand for secondary industries (manufacturing and construction) in emerging economies. Another way for the adjustment of industrial structure for decreasing emissions is to carry out internal adjustments in a specific industry, and especially manufacturing and construction, to speed up the transition to low-carbon industries such as high-end manufacturing. Adding technologies such as CCUS can increase industrial added value as well as reduce emissions.

In addition, adjusting the energy mix—through a gradual transition to renewable energy in the form of wind, solar, and hydro—is particularly important in regard to the secondary industry. The adjustment of the energy mix has long shown the decreasing effect of emissions in known developed countries, and the importance of renewable energy has also been estimated.^44^ For example, Le Quéré et al. found that the largest contribution to the reduction of emissions, about 36%–73%, came from reductions in the share of fossil fuels over the period 2005–2015 in 18 developed countries.^45^ Nevertheless, the energy transition in emerging economies is inadequate yet. The share of coal in Asia, such as in Laos and Cambodia, was still on the rise, which was one of the key factors contributing to the growth of emissions. For Tanzania and Myanmar, the declining share of biomass made the energy mix act as the driver for the reduction of emissions. Moreover, in India and South Africa, for instance, coal and oil dominated energy usage. Our study also showed that electricity and heat constituted the main source of CO_2_ emissions in most emerging economies, indicating that these emerging economies will take some time for emissions to peak and that they still have a long way to go in reducing emissions associated with the electricity sector. Thus, it is key to identify the reasons for the growth of emissions in emerging economies, especially by focusing on the substitution of energy use within the secondary industry (manufacturing and construction).^46^

Emerging economies may face investment challenges in the future. Under the guidance and support of policies, emerging economies need to actively attract foreign investment and adjust the flow of funds to the field of renewable energy. Meanwhile, there is substantial potential for promoting low-carbon technology transfer^47^ from developed countries to emerging economies to improve energy efficiency and reduce carbon intensity. COP26 brought to the forefront the divide between developed and developing nations, as the latter seek financial support to decouple economic development and emissions through renewable technologies. Given that emerging economies’ per-capita emissions are low from a climate justice perspective, there is a case to be made for developed nations to provide financial assistance and technology transfer to enable these economies to meet productive energy use goals.

Among sectors in Africa, the residential sector is responsible for most emissions from biomass combustion and also uses fossil fuels such as gas and coal. Thus, to tackle emission reduction in this sector in emerging economies, tools such as policies are needed to reduce the use of traditional biomass energy and fossil fuels and ease the adoption of electricity as much as possible.^48^^,^^49^^,^^50^ Decarbonization can be achieved through the displacement of traditional biomass such as deforestation for household cooking and fossil fuels by electrification. Meanwhile, the comparison of emission patterns and reduction factors at regional and national scales is more worthy of consideration. Latin America and the Caribbean have ample renewable energy, such as hydro and solar, which has been better utilized in recent years.^51^ Africa, with similar latitude, is also rich in renewable energy, but there is limited utilization. Therefore, the development experiences of renewable energy (considering opportunities for renewables investments and increasing the share of renewable energy while meeting the rapidly growing energy demand) in Latin America and the Caribbean,^52^ such as Brazil, Uruguay, and Paraguay, can be used to further explore renewable energy in Africa. Especially, renewable energy such as solar and hydro in Africa hereby optimize the energy structure and play a deserved role in reducing emissions. At the detailed country level, we still clearly see that coal increases Indonesia’s emissions and its use in electricity production. The key to reducing emissions lies in the more rational use of energy in all sectors, especially in electricity production to encourage the use of renewable energy such as geothermal energy (holding about 40% of the world^53^). Indonesia is an island country (with many islands), which makes economic development and energy distribution uneven. It is necessary to consider the energy distribution according to national circumstances and try to promote energy transformation by developing transport and the power grid.

In aggregate terms, accurate and refined data on emissions for emerging economies are the basis for the deployment of emission reduction strategies. Through data science, the effectiveness and causes of emission reduction are demonstrated more comprehensively. These 30 emerging economies selected for this study are representative to some extent. They include not only large emerging economies such as India and Brazil but also small and island emerging economies such as Jamaica. However, due to different geographical locations and resource endowments, the emission patterns of each emerging economy are heterogeneous, and potential energy transformation is inconsistent. The methodology used in this article is in accordance with the Intergovernmental Panel on Climate Change (IPCC) guidelines and is consistent with databases such as the IEA and the Global Carbon Budget (GCB). This article only considers energy-related emissions and does not account for industrial process emissions. Therefore, energy consumption in activity data and emission factors are the main sources of uncertainty. In particular, emission factors differ among emerging economies, and the availability of official emission factor information is limited. The accounting of energy-related emissions includes unsustainable biomass emissions, which is also one of the sources of uncertainty. In addition, the refinement of sectoral matching indicators will further improve the sector’s accounting results. We will continue to track the emission accounting and emission reduction potential of emerging economies in the future. More efforts are needed to conduct research and evaluate emerging economies.

### Methods

#### Carbon accounting

The compilation of emissions in emerging economies includes emissions related to energy use but excludes emissions from industrial processes. Accounting of CO_2_ emissions for emerging economies according to IPCC guidelines is shown in Equation 1:

(Equation 1) $$CE=\sum_{i}\sum_{j}CE_{ij}=\sum_{i}\sum_{j}AD_{ij}\times EF_{j,}$$

where *i* stands for industry and *j* represents the energy type including coal, oil, gas, and biomass. *CE*_*ij*_ is the carbon emissions of different industries and energy varieties. Given that our inventories are about emissions related to energy use, *AD*_*ij*_ is energy consumption data. *EF* is the emission factor, which can measure emissions by energy use per unit. Among them, in some emerging economies, the use of biomass is mainly sourced from wood and charcoal through deforestation, which is unsustainable over a certain period. Thus, the use of biomass in some emerging economies generates corresponding CO_2_ emissions in the process of accounting.

(Equation 2) $$EF_{j}=NCV_{j}\times CC_{j}\times O_{j}$$

The *EF* is obtained by multiplying the net calorific value (*NCV*), the carbon content (*CC*), and the oxidation rate (*O*), where *NCV* is the heat released by per unit of energy use, and the *CC* is the emissions per unit of heat. In addition, we also unify sectors of emerging economies according to some data or indicators, such as data of energy consumption or economic indicators. For a more comprehensive understanding, please refer to our published work, where we have presented detailed information.^54^

#### Index decomposition analysis

Index decomposition analysis (IDA) and structural decomposition analysis (SDA) have been used extensively to analyze the contributions of economic and social factors to changes of greenhouse gas and air pollutants in recent years.^55^ SDA needs to be deployed in combination with the input-output table, which reflects the interrelation among various sectors across countries in a certain period.^56^^,^^57^ Usually, the release time of the input-output table by institutions is discontinuous, which has a time lag. In contrast, IDA has relatively low data requirements and is relatively flexible on the selection of the time period studied. Moreover, IDA includes two other index methods, the Laspeyres^58^^,^^59^ and the Divisia.^60^ Given the characteristics of complete decomposition, convincing results, and theoretical basis, the accumulation method in the logarithmic mean division index (LMDI) is preferred in this study. We have used the LMDI method^61^^,^^62^ to analyze the changes in CO_2_ emissions of emerging economies. In this study, we denote CO_2_ emissions of emerging economies as follows:

(Equation 3) $$C=\sum_{i=1}^{3}\sum_{j=1}^{3}C_{ij}=\sum_{i=1}^{3}\sum_{j=1}^{3}\frac{C_{ij}}{E_{ij}}\times \frac{E_{ij}}{E_{i}}\times \frac{E_{i}}{G_{i}}\times \frac{G_{i}}{G}\times \frac{G}{P}\times P=\sum_{i=1}^{3}\sum_{j=1}^{3}CI_{\text{ij}}\times ES_{ij}\times EI_{ij}\times S_{i}\times Y\times P$$

This equation quantifies the total CO_2_ emissions from human sources as the product of several factors, building a bridge between emissions and important elements such as energy, economy, and population, where *i* represents industry, which is divided into the primary, secondary, and tertiary industries, and *j* represents the type of energy, which is divided into coal, oil, and gas. We decompose CO_2_ emissions into $CI_{ij}$, $ES_{ij}$, $EI_{ij}$, $S_{i}$, *Y*, and *P*. $CI_{ij}$ represents carbon intensity ($C_{ij}/E_{ij}$), which refers to the amount of CO_2_ emissions per unit of energy. $ES_{ij}$ represents the energy structure ($E_{ij}/E_{i}$), which describes the proportion of coal, oil, natural gas, biomass, and others in total energy. $EI_{ij}$ represents the energy intensity ($E_{i}/G_{i}$), which refers to the amount of energy required per unit of GDP. $S_{i}$ is the industrial structure ($G_{i}/G$), that is, the share of primary, secondary, and tertiary in total. *Y* is GDP per capita (G/P). *P* is population.

Specifically, we separately decompose CO_2_ emissions of the residential sector into the following factors:

(Equation 4) $$C^{r}=\sum_{j=1}^{3}C_{j}^{r}=\sum_{j=1}^{3}\frac{C_{j}^{r}}{E_{j}^{r}}\times \frac{E_{j}^{r}}{E^{r}}\times \frac{E^{r}}{P}\times P=\sum_{j=1}^{3}CI_{j}^{r}\times ES_{j}^{r}\times EP^{r}\times P$$

Similarly, in Equation 4, $C^{r}$ denotes carbon emissions in the residential sector. $CI_{j}^{r}$ represents the carbon intensity ($C_{j}^{r}/E_{j}^{r}$). $ES_{j}^{r}$ represents the energy structure ($E_{j}^{r}/E^{r}$). $EP^{r}$ represents energy per capita ($E^{r}/P$). *P* is population.

Thus, changes in CO_2_ emissions are decomposed into the following six factors over the specific time period.

(Equation 5) $$\Delta C=C_{t}-C_{0}=\Delta C_{CI}+\Delta C_{ES}+\Delta C_{EI}+\Delta C_{S}+\Delta C_{Y}+\Delta C_{P}$$

The contribution of each part in Equation 5 can be expressed as

(Equation 6) $$\Delta C_{CI}=\sum_{i=1}^{3}\sum_{j=1}^{3}\frac{C_{ij}^{t}-C_{ij}^{0}}{\ln\left(C_{ij}^{t}\right)-\ln\left(C_{ij}^{0}\right)}\times \ln\left(\frac{CI_{ij}^{t}}{CI_{ij}^{0}}\right)\text{,}$$

(Equation 7) $$\Delta C_{ES}=\sum_{i=1}^{3}\sum_{j=1}^{3}\frac{C_{ij}^{t}-C_{ij}^{0}}{\ln\left(C_{ij}^{t}\right)-\ln\left(C_{ij}^{0}\right)}\times \ln\left(\frac{ES_{ij}^{t}}{ES_{ij}^{0}}\right)\text{,}$$

(Equation 8) $$\Delta C_{EI}=\sum_{i=1}^{3}\sum_{j=1}^{3}\frac{C_{ij}^{t}-C_{ij}^{0}}{\ln\left(C_{ij}^{t}\right)-\ln\left(C_{ij}^{0}\right)}\times \ln\left(\frac{EI_{ij}^{t}}{EI_{ij}^{0}}\right)\text{,}$$

(Equation 9) $$\Delta C_{S}=\sum_{i=1}^{3}\sum_{j=1}^{3}\frac{C_{ij}^{t}-C_{ij}^{0}}{\ln\left(C_{ij}^{t}\right)-\ln\left(C_{ij}^{0}\right)}\times \ln\left(\frac{S_{i}^{t}}{S_{i}^{0}}\right)\text{,}$$

(Equation 10) $$\Delta C_{Y}=\sum_{i=1}^{3}\sum_{j=1}^{3}\frac{C_{ij}^{t}-C_{ij}^{0}}{\ln\left(C_{ij}^{t}\right)-\ln\left(C_{ij}^{0}\right)}\times \ln\left(\frac{Y^{t}}{Y^{0}}\right),$$

(Equation 11) $$\Delta C_{P}=\sum_{i=1}^{3}\sum_{j=1}^{3}\frac{C_{ij}^{t}-C_{ij}^{0}}{\ln\left(C_{ij}^{t}\right)-\ln\left(C_{ij}^{0}\right)}\times \ln\left(\frac{P^{t}}{P^{0}}\right)\text{.}$$

Similarly, CO_2_ emissions in the residential sector of emerging economies are analyzed by the LMDI method:

(Equation 12) $$\Delta C=C_{t}-C_{0}=\Delta C_{CI}+\Delta C_{ES}+\Delta C_{EP}+\Delta C_{P}$$

When accounting for biomass emissions in the decomposition analysis, the energy mix encompasses a range of sources, including coal, oil, gas, biomass, and others.

## Experimental procedures

### Resource availability

#### Lead contact

Further information should be directed to and will be fulfilled by the lead contact, Dabo Guan (guandabo@mail.tsinghua.edu.cn).

#### Materials availability

This study did not generate new unique materials.

## Data Availability

This study employs the energy data from the region’s or nation’s own statistics (Table S3) for CO_2_ emissions accounting. GDP and population data are from the United Nations,^63^ and GDP data are shown in US dollars (at constant 2015 prices). The data on CO_2_ emissions that support the findings of this study and the code of driving forces behind emissions change in this study can be seen from Zenodo^64^ (https://doi.org/10.5281/zenodo.7871178).

## Supplemental information

Document S1. Figures S1–S5 and Tables S1–S5

Document S2. Article plus supplemental information
