# Supplementary material for: The heterogeneous driving forces behind carbon emissions change in 30 selective emerging economies
Source: Patterns (N Y). 2023 May 30;4(7):100760. doi: 10.1016/j.patter.2023.100760 (PMC10382947; doi:10.1016/j.patter.2023.100760)
Supplement: Document S1. Figures S1–S5 and Tables S1–S5 [file mmc1.pdf]

Patterns, Volume *4*

Supplemental information

The heterogeneous driving forces behind carbon emissions change in 30 selective emerging economies

Shuping Li, Can Cui, Jing Meng, Yuan Li, Yuli Shan, Weichen Zhao, Priti Parikh, Jiawei Yao, and Dabo Guan

Supplemental Figures

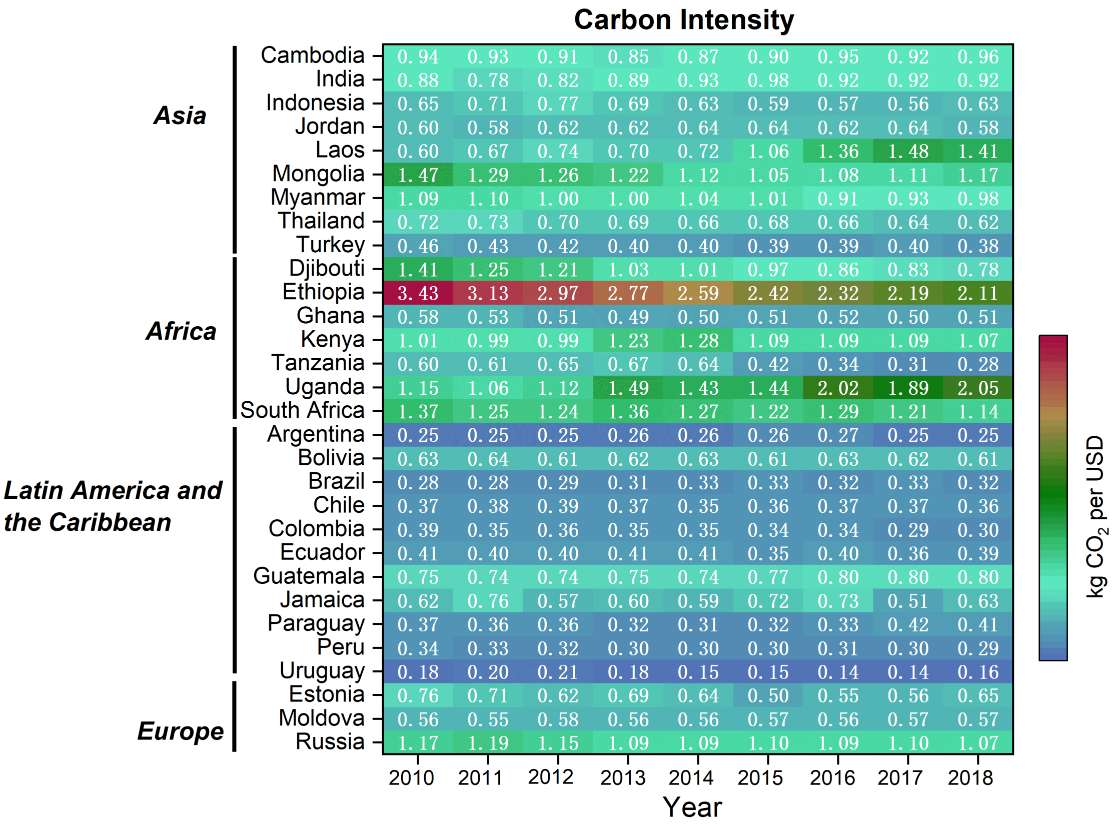

Figure S1. Carbon intensity (CO_2 emissions per GDP) in 30 selective emerging economies, 2010-2018.

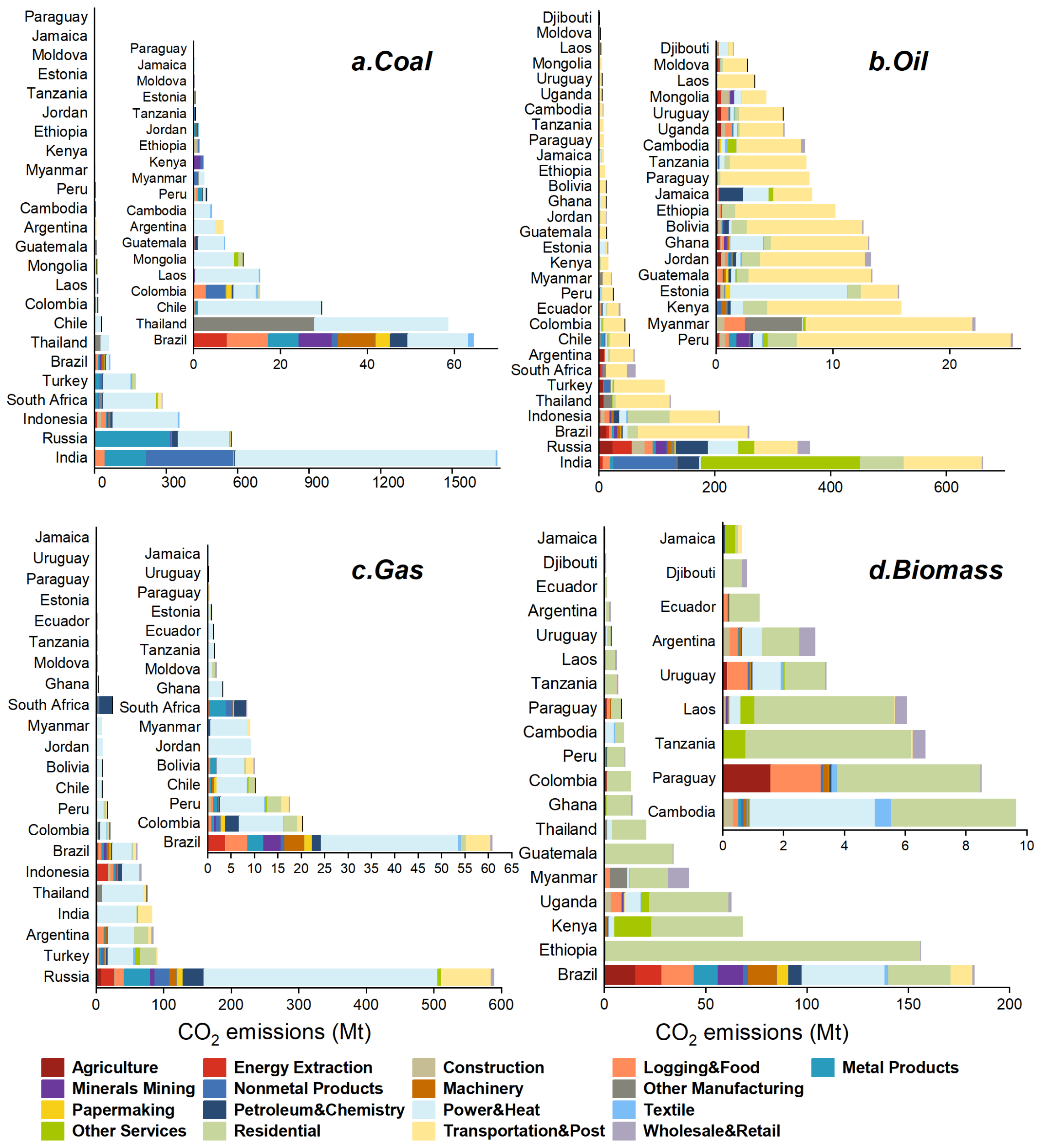

Figure S2. CO_2 emissions caused by different types of energy use, 2018.

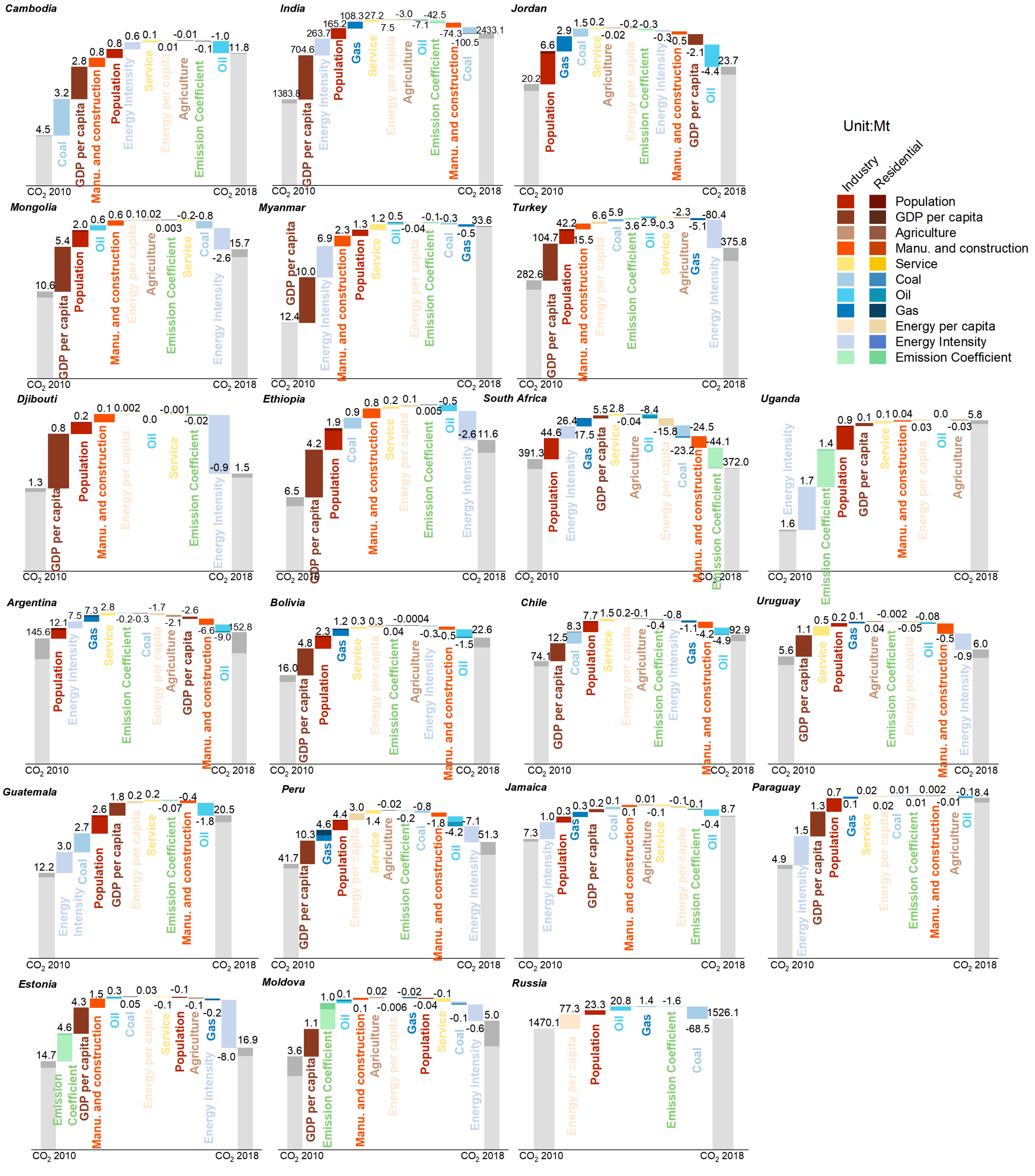

Figure S3. Drivers of CO_2 emissions changes about fossil fuels, 2010-2018.

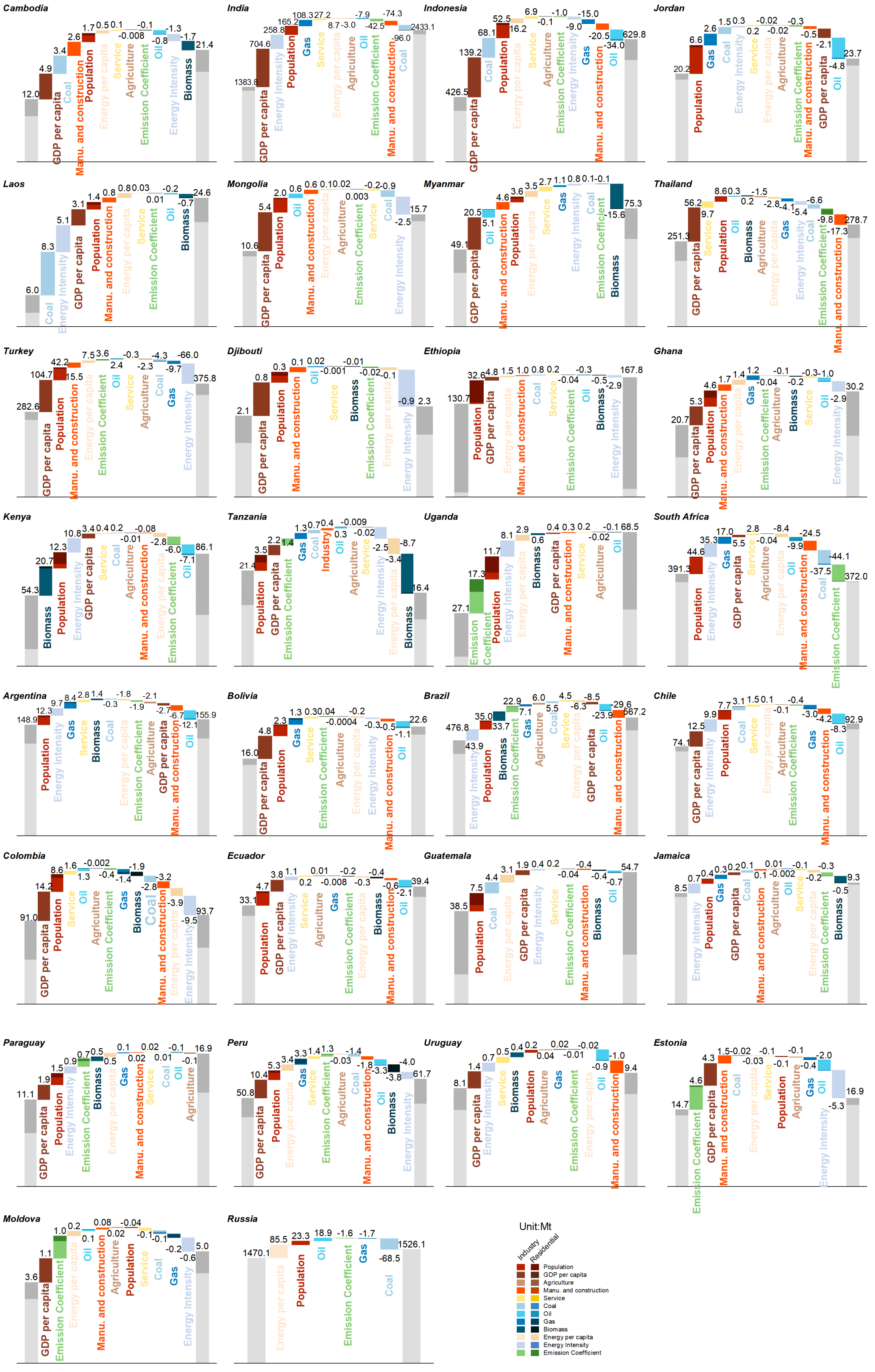

Figure S4. Drivers of CO_2 emissions changes including fossil fuels and biomass, 2010-2018. Among them, due to the availability of Russian, the industrial structure has not been analyzed. The decomposition factors in Russia only include emission coefficient, energy mix, energy per capita and population.

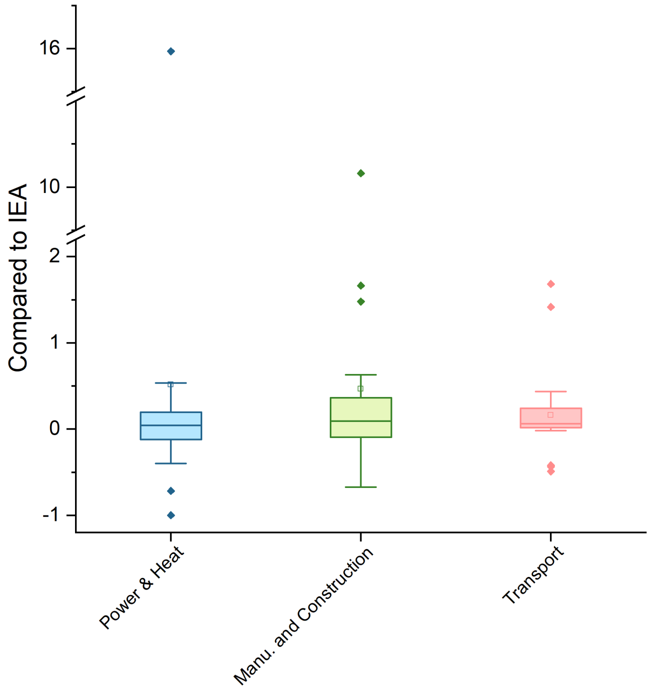

Figure S5. Data comparison with IEA about fossil fuels emissions among sectors in 2018.

Supplemental Tables

Table S1. Average annual growth rate of the secondary industry (manufacturing and construction) during 2010-2018 in 30 selective emerging economies, industrial structure in 2018, and net zero target.

	Country	Average annual growth rate of the manufacturing and construction	Agriculture	Manufacturing and construction	Service	Ranking	Net-zero target
The share of agriculture > The share of manufacturing and construction	Ethiopia	20.64%	34.15%	22.66%	43.18%	III>I>II	Yes
Average annual growth rate of manufacturing and construction < 5%	Uruguay	-1.93%	7.47%	20.24%	72.29%	III>II>I	Yes
	Brazil	-0.88%	5.51%	21.59%	72.90%	III>II>I	Yes
	Argentina	-0.51%	5.20%	26.16%	68.63%	III>II>I	Yes
	South Africa	0.63%	2.36%	28.36%	69.28%	III>II>I	Yes
	Jamaica	1.10%	7.84%	22.55%	69.61%	III>II>I	Yes
	Thailand	1.84%	8.64%	34.56%	56.81%	III>II>I	Yes
	Russia	1.84%	4.30%	33.77%	61.93%	III>II>I	Yes
	Jordan	2.04%	5.15%	27.38%	67.47%	III>II>I	No
	Chile	2.05%	4.02%	31.59%	64.39%	III>II>I	Yes
	Colombia	2.50%	6.84%	29.62%	63.54%	III>II>I	Yes
	Ecuador	2.60%	10.69%	32.75%	56.56%	III>II>I	Yes
	Guatemala	2.87%	10.56%	23.15%	66.29%	III>II>I	No
	Peru	3.15%	7.97%	32.66%	59.36%	III>II>I	Yes
	Bolivia	4.24%	13.03%	29.42%	57.55%	III>II>I	No
	Paraguay	4.28%	9.97%	37.30%	52.73%	III>II>I	No
	Indonesia	4.48%	13.51%	40.58%	45.91%	III>II>I	Yes
	Moldova	4.71%	15.23%	25.78%	58.99%	III>II>I	No
	Kenya	4.97%	19.17%	19.21%	61.62%	III>II>I	No
	Uganda	4.58%	22.77%	29.99%	47.23%	III>II>I	No
Average annual growth rate of manufacturing and construction > 5%	Estonia	5.30%	1.94%	29.15%	68.91%	III>II>I	Yes
	India	5.75%	17.05%	29.65%	53.30%	III>II>I	Yes
	Turkey	7.32%	7.06%	31.53%	61.41%	III>II>I	Yes
	Mongolia	8.62%	14.73%	32.81%	52.46%	III>II>I	No
	Tanzania	9.28%	27.67%	29.27%	43.07%	III>II>I	Yes
	Myanmar	9.40%	22.29%	36.04%	41.67%	III>II>I	Yes
	Laos	9.69%	17.51%	34.78%	47.71%	III>II>I	Yes
	Ghana	10.94%	21.05%	38.17%	40.78%	III>II>I	Yes
	Djibouti	11.67%	1.52%	17.30%	81.18%	III>II>I	No
	Cambodia	11.94%	24.06%	33.70%	42.24%	III>II>I	Yes

Table S2 List of Sectoral classification.

No.	Sector_47	Sector_17
**1**	Agriculture, forestry, hunting, fishing and husbandry	Agriculture, forestry, hunting, fishing and<br>husbandry
**2**	Coal Mining and Dressing	Coal Mining, Petroleum and Natural Gas<br>Extraction
**3**	Petroleum and Natural Gas Extraction	
**4**	Ferrous Metals Mining and Dressing	Minerals Mining and Dressing
**5**	Nonferrous Metals Mining and Dressing	
**6**	Nonmetal Minerals Mining and Dressing	
**7**	Other Minerals Mining and Dressing	
**8**	Logging and Transport of Wood and Bamboo	Logging, Timber, Food, Beverage and Tobacco
**9**	Food Processing	
**10**	Food Production	
**11**	Beverage Production	
**12**	Tobacco Processing	
**13**	Timber Processing, Bamboo, Cane, Palm Fiber & Straw Products	
**14**	Furniture Manufacturing	
**15**	Textile Industry	Textile, Garments and Leather
**16**	Garments and Other Fiber Products	
**17**	Leather, Furs, Down and Related Products	
**18**	Papermaking and Paper Products	Papermaking, Printing and Cultural
**19**	Printing and Record Medium Reproduction	
**20**	Cultural, Educational and Sports Articles	
**21**	Petroleum Processing and Coking	Petroleum Processing, Raw Chemical, and<br>Medical
**22**	Raw Chemical Materials and Chemical Products	
**23**	Medical and Pharmaceutical Products	
**24**	Chemical Fiber	
**25**	Rubber Products	Nonmetal Mineral Products
**26**	Plastic Products	
**27**	Nonmetal Mineral Products	
**28**	Smelting and Pressing of Ferrous Metals	Metal Smelting, Pressing and Products
**29**	Smelting and Pressing of Nonferrous Metals	
**30**	Metal Products	
**31**	Ordinary Machinery	Ordinary, Special, Transportation, Electric,<br>Electronic and Instrument Machinery
**32**	Equipment for Special Purposes	
**33**	Transportation Equipment	
**34**	Electric Equipment and Machinery	
**35**	Electronic and Telecommunications	

	Equipment	
**36**	Instruments, Meters, Cultural and Office Machinery	
**37**	Other Manufacturing Industry	Other Manufacturing and Waste
**38**	Scrap and waste	
**39**	Production and Supply of Electric Power, Steam and Hot Water	Production of Electricity, Heat, Gas and Tap Water
**40**	Production and Supply of Gas	
**41**	Production and Supply of Tap Water	
**42**	Construction	Construction
**43**	Transportation, Storage, Post and Telecommunication Services	Transportation, Storage, Post and Telecommunication Services
**44**	Wholesale, Retail Trade and Catering Services	Wholesale, Retail Trade and Catering Services
**45**	Other Services	Other Services
**46**	Urban	Residential
**47**	Rural	

Data sources

Table S3 Data sources of energy.

Country	Website
Argentina	https://www.argentina.gob.ar/economia/energia/hidrocarburos/balances-energeticos
Bolivia	https://www.hidrocarburos.gob.bo/
Brazil	https://www.epe.gov.br/sites-pt/publicacoes-dados-abertos/publicacoes/PublicacoesArquivos/publicacao-377/topico-494/BEN%202019%20Completo%20WEB.pdf
Chile	http://energiaabierta.cl/visualizaciones/balance-de-energia/
Colombia	https://www1.upme.gov.co/InformacionCifras/Paginas/BECOCONSULTA.aspx
Djibouti	https://au-afrec.org/
Ecuador	https://www.recursosyenergia. gob.ec/
Estonia	http://pub.stat.ee/px-web.2001/I_Databas/Economy/07Energy/02Energy_consumption_and_production/01Annual_statistics/01Annual_statistics.asp
Ethiopia	http://www.csa.gov.et/
Ghana	http://www.energycom.gov.gh/files
Guatemala	https://mem.gob.gt/
Jordan	https://www.memr.gov.jo/Default/Ar
Kenya	https://unstats.un.org/unsd/energystats/pubs/balance/
Cambodia	https://www.eria.org/RPR_FY2015_No.8_Chapter_2.pdf
Laos	https://www.eria.org/publications/energy-demand-and-supply-of-the-lao-peoples-democratic-republic-2010-2018/
Indonesia	https://www.bps.go.id/

Moldova	https://statbank.statistica.md/PxWeb/pxweb/ro/40%20Statistica%20economica/40%20Statistica%20economica__15%20ENE__serii%20anuale/ENE020100.px/?rxid=b2ff27d7-0b96-43c9-934b-42e1a2a9a774
Myanmar	https://www.eria.org/publications/energy-demand-and-supply-of-the-republic-of-the-union-of-myanmar-2010-2017/
Mongolia	https://www.1212.mn/Stat.aspx?LIST_ID=976_L11&type=tables
Peru	https://sinia.minam.gob.pe/
Paraguay	http://www.dgeec.gov.py/
Thailand	https://www.dede.go.th/ewt_news.php?nid=47340
Turkey	https://www.dunyaenerji.org.tr/turkiye-enerji-denge-tablolari/
Tanzania	https://au-afrec.org/
Uganda	https://au-afrec.org/
Uruguay	https://www.ine.gub.uy/inicio
Jamaica	https://www.mset.gov.jm/document-category/energy-balances/
Russia	https://fedstat.ru/indicator
India	http://mospi.gov.in/
South Africa	http://www.energy.gov.za/files/media/Energy_Balances.html

Data comparison

Table S4. Data comparison with IEA about fossil fuels emissions. Unit: million tons.

Country	Year	IEA	CEADs
Argentina	2010	162.4	145.6
	2011	171.2	155.4
	2012	172.6	156.7
	2013	177.4	162.8
	2014	173.9	159.8
	2015	179.8	166.0
	2016	178.1	165.4
	2017	173.9	161.9
	2018	171.2	152.8
Bolivia	2010	13.6	16.0
	2011	14.8	17.0
	2012	15.8	17.0
	2013	16.8	18.5
	2014	18.1	19.8
	2015	18.5	20.0
	2016	19.8	21.5
	2017	20.2	22.1
	2018	20.3	22.6
Brazil	2010	373.2	360.3
	2011	392.2	378.5
	2012	425.5	410.5
	2013	455.6	439.2

	2014	479.7	453.6
	2015	455.9	431.6
	2016	420.1	395.9
	2017	430.0	406.6
	2018	408.1	384.6
Chile	2010	68.6	74.1
	2011	75.3	82.3
	2012	77.2	86.8
	2013	81.9	85.9
	2014	75.5	82.8
	2015	81.1	87.0
	2016	85.3	91.3
	2017	86.2	92.8
	2018	85.8	92.9
Colombia	2010	59.2	73.4
	2011	63.9	71.1
	2012	63.5	76.1
	2013	71.5	79.5
	2014	73.4	83.8
	2015	74.1	83.8
	2016	77.2	86.8
	2017	69.5	75.5
	2018	73.2	80.5
Djibouti	2010	0.5	1.3
	2011	0.5	1.3
	2012	0.5	1.5
	2013	0.5	1.4
	2014	0.3	1.4
	2015	0.4	1.4
	2016	0.3	1.5
	2017	0.3	1.5
	2018	0.3	1.5
Ecuador	2010	31.6	31.5
	2011	32.1	33.4
	2012	33.3	34.9
	2013	35.7	37.7
	2014	37.4	39.6
	2015	37.2	33.4
	2016	35.5	37.5
	2017	34.4	34.7
	2018	36.4	38.2
Estonia	2010	18.2	14.7
	2011	18.1	14.7

	2012	16.6	13.2
	2013	18.0	15.2
	2014	16.9	14.5
	2015	14.0	11.4
	2016	15.5	12.9
	2017	16.3	14.0
	2018	15.2	16.9
Ethiopia	2010	5.8	6.5
	2011	6.8	6.7
	2012	7.2	7.7
	2013	8.4	8.4
	2014	10.4	9.4
	2015	10.1	10.5
	2016	12.0	10.9
	2017	12.4	11.2
	2018	13.3	11.6
Ghana	2010	10.3	10.6
	2011	10.7	11.0
	2012	12.8	11.5
	2013	13.7	12.0
	2014	13.1	12.6
	2015	14.0	13.1
	2016	14.2	13.7
	2017	14.5	14.9
	2018	16.4	16.4
Guatemala	2010	10.4	12.2
	2011	10.7	12.5
	2012	11.1	13.1
	2013	11.9	14.3
	2014	12.6	15.1
	2015	15.0	18.3
	2016	16.1	19.7
	2017	15.2	19.0
	2018	16.9	20.5
Jordan	2010	18.8	20.2
	2011	19.8	20.0
	2012	22.7	22.1
	2013	22.3	22.6
	2014	24.0	24.2
	2015	23.7	24.5
	2016	23.1	24.4
	2017	24.4	25.9
	2018	23.0	23.7

Kenya	2010	11.6	10.3
	2011	11.7	10.7
	2012	10.7	11.1
	2013	11.9	12.0
	2014	13.8	13.6
	2015	14.9	16.0
	2016	16.0	16.7
	2017	17.0	17.3
	2018	16.2	18.0
Cambodia	2010	4.8	4.5
	2011	4.9	5.2
	2012	5.2	5.4
	2013	5.2	5.2
	2014	6.4	6.2
	2015	7.8	7.4
	2016	9.6	9.7
	2017	10.4	10.4
	2018	10.2	11.8
Laos	2010	2.4	1.8
	2011	2.6	2.1
	2012	2.7	2.5
	2013	3.3	2.8
	2014	3.5	3.6
	2015	7.7	9.2
	2016	14.4	14.9
	2017	17.6	18.3
	2018	17.8	18.6
Indonesia	2010	391.7	426.5
	2011	450.3	497.2
	2012	451.4	573.5
	2013	417.3	538.2
	2014	452.9	515.5
	2015	458.6	508.1
	2016	449.7	517.2
	2017	481.7	534.8
	2018	533.0	629.8
Moldova	2010	7.9	3.6
	2011	7.9	3.8
	2012	7.7	4.0
	2013	6.7	4.1
	2014	7.2	4.3
	2015	7.6	4.4
	2016	7.7	4.6

	2017	7.5	4.8
	2018	8.0	5.0
Myanmar	2010	7.9	12.4
	2011	8.5	13.2
	2012	11.6	12.0
	2013	13.2	12.9
	2014	16.5	17.3
	2015	18.7	22.6
	2016	20.9	21.2
	2017	30.4	29.7
	2018	30.3	33.6
Mongolia	2010	14.1	10.6
	2011	15.6	11.0
	2012	17.0	12.0
	2013	18.3	13.0
	2014	17.9	12.8
	2015	17.1	12.4
	2016	18.0	12.8
	2017	19.3	14.0
	2018	21.1	15.7
Peru	2010	41.5	41.7
	2011	44.9	42.7
	2012	44.1	45.0
	2013	45.2	45.6
	2014	48.1	46.9
	2015	49.5	48.3
	2016	52.2	52.5
	2017	49.8	50.8
	2018	50.1	51.3
Paraguay	2010	4.8	4.9
	2011	5.0	5.1
	2012	4.9	5.3
	2013	5.1	5.2
	2014	5.4	5.5
	2015	6.0	6.1
	2016	6.8	6.9
	2017	7.7	8.0
	2018	8.1	8.4
Thailand	2010	221.9	219.6
	2011	220.1	223.9
	2012	236.6	228.4
	2013	245.0	232.9
	2014	240.9	221.6

	2015	248.0	237.7
	2016	244.1	252.5
	2017	244.1	254.3
	2018	241.4	258.0
Turkey	2010	267.8	282.6
	2011	287.2	292.1
	2012	298.4	302.1
	2013	285.2	312.3
	2014	307.2	322.9
	2015	318.8	333.9
	2016	338.6	349.0
	2017	378.3	382.3
	2018	369.4	375.8
Tanzania	2010	6.0	6.1
	2011	7.1	7.8
	2012	8.6	9.9
	2013	9.8	10.2
	2014	9.0	10.2
	2015	9.6	11.4
	2016	9.1	10.4
	2017	10.0	10.1
	2018	10.3	9.7
Uganda	2010	3.0	1.6
	2011	3.3	3.3
	2012	3.3	2.8
	2013	3.3	3.8
	2014	3.7	4.1
	2015	4.4	4.4
	2016	4.6	4.7
	2017	4.8	4.8
	2018	5.5	5.8
Uruguay	2010	6.0	5.6
	2011	7.2	7.0
	2012	8.2	7.9
	2013	7.0	6.8
	2014	6.2	5.9
	2015	6.3	6.1
	2016	6.2	6.0
	2017	5.8	5.9
	2018	6.2	6.0
Jamaica	2010	7.2	7.3
	2011	7.2	9.6
	2012	6.6	7.1

	2013	7.0	7.4
	2014	6.8	7.2
	2015	6.7	9.2
	2016	7.2	9.8
	2017	6.9	6.9
	2018	8.3	8.7
Russia	2010	1505.3	1470.1
	2011	1580.4	1553.7
	2012	1583.1	1565.9
	2013	1543.3	1504.6
	2014	1525.8	1510.5
	2015	1504.6	1502.7
	2016	1478.5	1495.4
	2017	1506.3	1526.3
	2018	1566.7	1526.4
India	2010	1571.5	1383.8
	2011	1661.8	1284.6
	2012	1804.4	1434.8
	2013	1860.1	1638.2
	2014	2026.3	1846.1
	2015	2034.7	2098.4
	2016	2066.5	2134.2
	2017	2183.4	2290.7
	2018	2315.3	2433.1
South Africa	2010	420.3	391.3
	2011	404.7	366.6
	2012	422.3	373.2
	2013	432.3	418.8
	2014	443.3	397.8
	2015	419.6	386.9
	2016	419.9	410.4
	2017	429.7	390.1
	2018	434.1	372.0

Table S5. Data comparison with IEA about fossil fuels emissions by sectors in 2018. Unit: million tons.

	IEA	CEADs	IEA	CEADs	IEA	CEADs
	Power and Heat	Power and Heat	Manu. and Construction	Manu. and Construction	Transport	Transport
Argentina	47.07	48.73	20.72	19.72	47.79	47.13
Bolivia	4.00	6.13	2.25	3.20	10.71	11.49
Brazil	62.32	51.87	89.03	104.51	191.87	194.65
Chile	32.95	36.37	14.33	15.77	28.33	32.32

Colombia	13.01	16.81	15.72	19.31	29.34	37.16
Djibouti	0.05	0.80	0.02	0.26	0.14	0.37
Ecuador	5.78	6.40	3.04	7.53	19.16	20.17
Estonia	11.43	10.77	0.66	1.1	2.46	3.17
Ethiopia	0.00	0.00	4.89	1.99	6.84	8.50
Ghana	5.19	5.95	1.94	1.14	8.06	8.31
Guatemala	5.27	6.36	1.94	2.65	8.56	10.52
Jordan	9.02	9.66	1.62	2.55	9.01	8.98
Kenya	1.46	1.04	2.99	3.42	9.67	11.41
Cambodia	3.42	4.14	0.78	1.05	5.12	5.50
Laos	14.04	14.76	0.63	0.65	3.12	3.11
Indonesia	217.00	311.53	112.07	157.10	150.99	84.99
Moldova	3.26	0.92	1.08	0.35	2.10	2.16
Myanmar	7.61	9.09	8.48	9.42	6.10	14.73
Mongolia	13.55	9.86	1.96	1.58	2.17	2.13
Peru	10.94	10.75	8.12	8.50	24.18	25.30
Paraguay	0.00	0.00	0.22	0.13	7.61	7.97
Thailand	87.39	92.57	48.17	52.12	72.11	98.18
Turkey	144.22	156.42	72.69	72.53	83.37	86.16
Tanzania	2.74	1.65	1.94	1.11	5.06	6.45
Uganda	0.23	0.30	1.17	1.10	3.39	3.73
Uruguay	0.32	0.32	0.91	0.89	3.63	3.81
Jamaica	2.79	2.45	2.82	2.55	2.25	3.23
Russia	815.80	615.12	250.55	667.36	258.86	149.85
India	1197.16	1155.07	565.29	767.76	305.33	155.06
South Africa	233.28	220.68	54.24	73.03	55.60	50.92

CO_2 emissions about fossil fuels different from IEA for interquartile ranges covering 50% of the data ranging from -12.2%-19.4% for electricity and heat production, 9.4%-36.1% for manu. industries and construction, and 1.5%-24.3% for transport (Figure S5 and Table S5). The outliers observed in the sectors of Power & Heat and Manu. and Construction in Figure S5 can be attributed to different CO_2 emission for Djibouti. To quantify CO_2 emissions of Djibouti, the energy data used in this study were primarily sourced from the energy balance tables published on the African Energy Commission (AFREC). In contrast, the information of energy balance for Djibouti is not published in the IEA. The CO_2 emissions of Djibouti published in the Greenhouse Gas Emissions from Energy database by the IEA are estimated using data from the United Nations Statistics Division (UNSD) 2017 Energy Balances publication.

In addition, the IEA provides emissions of other and biofuels and renewable wastes. Other emissions include industrial waste and non-renewable municipal waste emissions. Biofuels and renewable wastes include biofuels (primary solid biofuels, biogases, biogasoline, biodiesels, bio jet kerosene and other liquid biofuels) and renewable wastes. However, biofuels and renewable wastes emissions are not counted in the total emissions. Due to the different ways of consideration, we divide biomass into

sustainable and unsustainable. For sustainable biomass we do not consider its emissions, while for unsustainable biomass we consider its emissions. Sustainable biomass, including bagasse, biogas and biodiesel, is considered green throughout whole life cycle and in line with the perspective of IEA. As such, we don't factor in emissions related to the use of sustainable biomass. Obtaining firewood and charcoal by logging not only degrades forests but also has a detrimental impact on the environment, which is not sustainable, especially in Africa. Thus, we have included this segment of emissions which is similar to the charcoal and primary solid biomass studied by the IEA. It is worth noting that the primary solid biomass we have examined has a narrower scope of coverage compared to the IEA's analysis. For example, we don't consider animal dropping emissions.
