# Supplementary material for: The heterogeneous driving forces behind carbon emissions change in 30 selective emerging economies
Source: Patterns (N Y). 2023 May 30;4(7):100760. doi: 10.1016/j.patter.2023.100760 (PMC10382947; doi:10.1016/j.patter.2023.100760)
Supplement: Document S2. Article plus supplemental information [file mmc2.pdf]

Patterns

Article

The heterogeneous driving forces behind carbon emissions change in 30 selective emerging economies

Graphical abstract

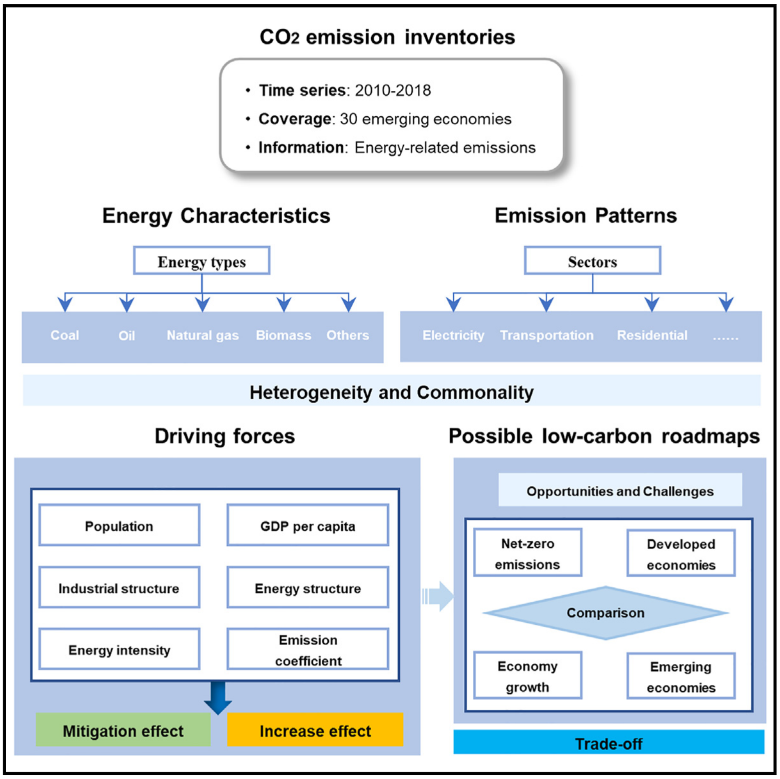

Authors

Shuping Li, Can Cui, Jing Meng, ..., Priti Parikh, Jiawei Yao, Dabo Guan

Correspondence

jing.j.meng@ucl.ac.uk (J.M.), guandabo@mail.tsinghua.edu.cn (D.G.)

In brief

Emerging economies are crucial to mitigating global climate change and reducing CO_2 emissions in the future. Through the comprehensive analysis of emission patterns and the identification of potential reduction factors in emerging economies, we can provide enhanced support for the deployment and execution of their policies.

Highlights

- CO_2 emissions and economic growth in 30 selective emerging economies are estimated
- Energy intensity impels emissions to decrease, showing a large reduction potential
- Changes in the energy mix and industrial structure affect emissions decline
- The heterogeneity of energy use and sectoral emissions has emerged in economies

Li et al., 2023, Patterns *4*, 100760
July 14, 2023 © 2023 The Authors.
https://doi.org/10.1016/j.patter.2023.100760

CellPress

Patterns

CellPress
OPEN ACCESS

Article

The heterogeneous driving forces behind carbon emissions change in 30 selective emerging economies

Shuping Li,[1] Can Cui,[2] Jing Meng,[3,*] Yuan Li,[1,3] Yuli Shan,[4] Weichen Zhao,[3] Priti Parikh,[3] Jiawei Yao,[5] and Dabo Guan[2,3,6,*]
[1]Institute of Blue and Green Development, Weihai Institute of Interdisciplinary Research, Shandong University, Weihai 264209, China
[2]Department of Earth System Sciences, Tsinghua University, Beijing 100080, China
[3]The Bartlett School of Sustainable Construction, University College London, London WC1E 7HB, UK
[4]School of Geography, Earth and Environmental Sciences, University of Birmingham, Birmingham B15 2TT, UK
[5]College of Architecture and Urban Planning, Tongji University, Shanghai 200092, China
[6]Lead contact
*Correspondence: jing.j.meng@ucl.ac.uk (J.M.), guandabo@mail.tsinghua.edu.cn (D.G.)
https://doi.org/10.1016/j.patter.2023.100760

THE BIGGER PICTURE Increasing attention has been paid to CO_2 emissions, which are the main source of greenhouse gas emissions (GHGs) and are closely related to temperature rise in climate change. Growing CO_2 emissions and ambiguous low-carbon transition paths have gradually become the bright spot in emerging economies. However, due to insufficient data, our understanding of which factors are the most effective drivers of emission reduction in emerging economies remains limited. Accordingly, we try to evaluate the disparity of emission trajectories in 30 selective emerging economies according to compiled emission accounts and find the heterogeneity of sector emissions and key drivers of changes in emissions to provide some thoughts for future emission reduction policies of emerging economies.

1 2 3 4 5 **Proof-of-Concept:** Data science output has been formulated, implemented, and tested for one domain/problem

SUMMARY

Emerging economies are predicted to be future emission hotspots due to expected levels of urbanization and industrialization, and their CO_2 emissions are receiving more scrutiny. However, the driving forces underlying dynamic change in emissions are poorly understood, despite their crucial role in developing targeted mitigating pathways. We firstly compile energy-related emissions of 30 selective emerging economies from 2010 to 2018. Then, three growth patterns of emissions in these economies have been identified through emission data, which imply different low-carbon pathways. Most emerging economies saw an increase of varying degrees in emissions, driven by economic growth and partly offset by better energy efficiency and improvements in energy mixes. Furthermore, the industrial structure was another factor that slowed emissions, especially in Latin America and the Caribbean. Our research contributes to the heterogeneous exploration of CO_2 emissions produced by energy among sectors and the creation of low-carbon development pathways in emerging economies.

INTRODUCTION

To slow the progress of global climate change, nations are increasingly setting carbon reduction targets toward carbon neutrality. Most developed countries reached their peak emissions point before 2020[1] and are pursuing energy transitions involving renewables and decarbonization.[2,3] In contrast, the rapid industrialization and urbanization rolling out in emerging economies mean that emissions have been increasing rapidly in recent years, which presents a challenge to global climate change mitigation.[4–7] While the per-capita emissions in emerging countries are much lower than the level associated with the 1.5°C (i.e., the Paris Agreement identifies efforts to control global temperature rise to no more than 1.5°C above pre-industrial levels by

This is an open access article under the CC BY license (http://creativecommons.org/licenses/by/4.0/).

2100) goal,[8] it is likely that per-capita emissions in emerging economies will further rise in tandem with economic growth.[9–11] The challenge facing emerging economies lies in achieving net-zero targets while simultaneously meeting the socioeconomic needs of their citizens. Therefore, trends and patterns in CO_2 emissions and related driving factors in developing countries need investigation to add detail and nuance to strategies for low-carbon transitions.

A series of studies have analyzed emission patterns and driving forces in emerging economies, although at an aggregated regional level.[12,13] The aggregated national CO_2 emissions to the region level mask the heterogeneity in emissions across countries. Some recent studies have focused on individual emerging countries such as China,[14,15] India,[16–18] Malaysia,[19] Turkey,[20] and Ethiopia.[21] However, it is difficult to compare the changes in emission levels and driving forces across countries due to the different data sources and time periods across studies. Recently, a few studies analyzed drivers of CO_2 emissions in a group of developing countries. Steckel et al., by studying 20 sub-Saharan African nations, found the drivers to include population, per-capita gross domestic product (GDP), energy intensity, and carbon intensity of CO_2 emissions and highlighted the role of rising carbon intensity in increasing emissions.[22] Similarly, Ayompe et al., by analyzing emissions from the combustion of fossil fuels in 27 African countries, found that population and GDP were driving the growth of emissions.[9] However, these studies focused on single regions of emerging economies and did not reveal the impact of economic structure related to emission patterns and trends, making CO_2 emissions across regions with heterogeneous economic structures difficult to compare. More importantly, the pressure to reduce emissions varies among sectors, as do their reduction pathways. Owing to the need for socioeconomic development and coping with climate change, the transition from coal to renewable energy is, for example, deployed in the power sector,[23,24] but the transition from fossil fuel or traditional biomass to clean fuel,[25] as well as factors affecting CO_2 emissions such as building area and income,[26–28] are seen as taking place in the residential sector. It is thus necessary to find the key high-emission industries as well as energy with high carbon content in emerging economies before targeted emission reduction plans are formulated. Moreover, given the significant disparities in levels of economic and technological development among emerging economies, there is significant spatiotemporal heterogeneity in their energy structures, energy efficiencies, industrial structures, and CO_2 emissions,[10,29] which need to be analyzed one by one.

This study therefore first quantifies the driving factors of various CO_2 emission trajectories in 30 selective emerging economies over the period from 2010 to 2018. On the one hand, fossil energy-related CO_2 emissions in the 30 countries accounted for about 19.8% of the world's total emissions, 1.4 times that of the United States and over 60% of China's emissions in 2018, according to the International Energy Agency (IEA).[30] On the other hand, the population growth rate in these emerging economies is much faster than in developed countries such as the United States. According to the World Bank,[31] 24 of the 30 countries have population growth rates well above the 0.5% and 0.6% rates of the United States and United Kingdom, with Uganda, Tanzania, and Ethiopia experiencing population growth of up to 3.4%, 3.2%, and 2.7%, respectively. Rapid population growth signifies that these countries face higher energy demand than developed countries such as the United States and the United Kingdom, which poses challenges and risks for these countries to work toward energy transformation and emission reduction. Thus, emission patterns and potential emission reduction factors in these countries need to be mapped as soon as possible. We have used our newly built CO_2 emission inventories, which enable transparent and comparable analysis. Thereafter, this study discusses the possible low-carbon roadmaps for these countries. This study distinguishes the heterogeneity of emissions and provides quantitative evidence for policy formulation to mitigate climate change in emerging economies.

RESULTS

Economic development and national CO_2 emission trends

The relative growth of CO_2 emissions and GDP (in 2018 compared with 2010) in the 30 emerging economies can be divided into three groups, as demonstrated in quadrant IV and two areas divided by a dashed gray line in quadrant I (Figure 1). South Africa and Tanzania (group one) have shown a downward trend in CO_2 emissions and an upward trend in GDP (quadrant IV). In 2018, the GDP per capita values of South Africa and Tanzania were $5,636 and $1,052, respectively. In South Africa, CO_2 emissions decreased by 5% from 2010 (391.3 Mt) to 2018 (372 Mt), while its economy grew by 14.4% compared with 2010—from $284.7 billion to $325.7 billion. In Tanzania, CO_2 emissions decreased by 23.2% from 21.4 (2010) to 16.4 Mt (2018), while its GDP has grown by 67.4% from $35.4 billion to $59.3 billion. These unusual developments, of lower emissions and rising GDP, might be an exception, or South Africa and Tanzania might see emissions rebound in the future. The population of Africa is growing rapidly, which will lead to a demand for increased energy consumption for domestic, industrial, and food production needs. Meanwhile, energy access is also a challenge on the continent.[32] About 580 million people in sub-Saharan Africa had no access to electricity in 2019,[33] and the situation is likely to worsen under the COVID-19 pandemic. Electricity consumption and associated emissions would be highly likely to increase rapidly with the development of electricity infrastructure and an increase in energy use unless there is a rapid acceleration of investment in off-grid renewable technologies.[34] Thus, although CO_2 emissions in South Africa and Tanzania have declined markedly, these countries may not be able to maintain this trend in the future.

Emerging economies in the other two groups witnessed rising CO_2 emissions and GDP. Those countries in group two have a lower growth rate of CO_2 emissions than of GDP, which is located between the horizontal dashed line and the gray dashed line in quadrant I of Figure 1. For example, Uruguay's rate of change in CO_2 emissions was 15.7% in 2010–2018, which was lower than the rate of economic growth (33.2%). Similarly, the economies of Ethiopia and Djibouti developed rapidly, pushing the economic growth rate to more than 3.8 and 8.8 times that of CO_2 emissions. The level of economic development and the GDP per capita ($19,655 in 2018) of Estonia is relatively high. The range of change in economic growth in Estonia was

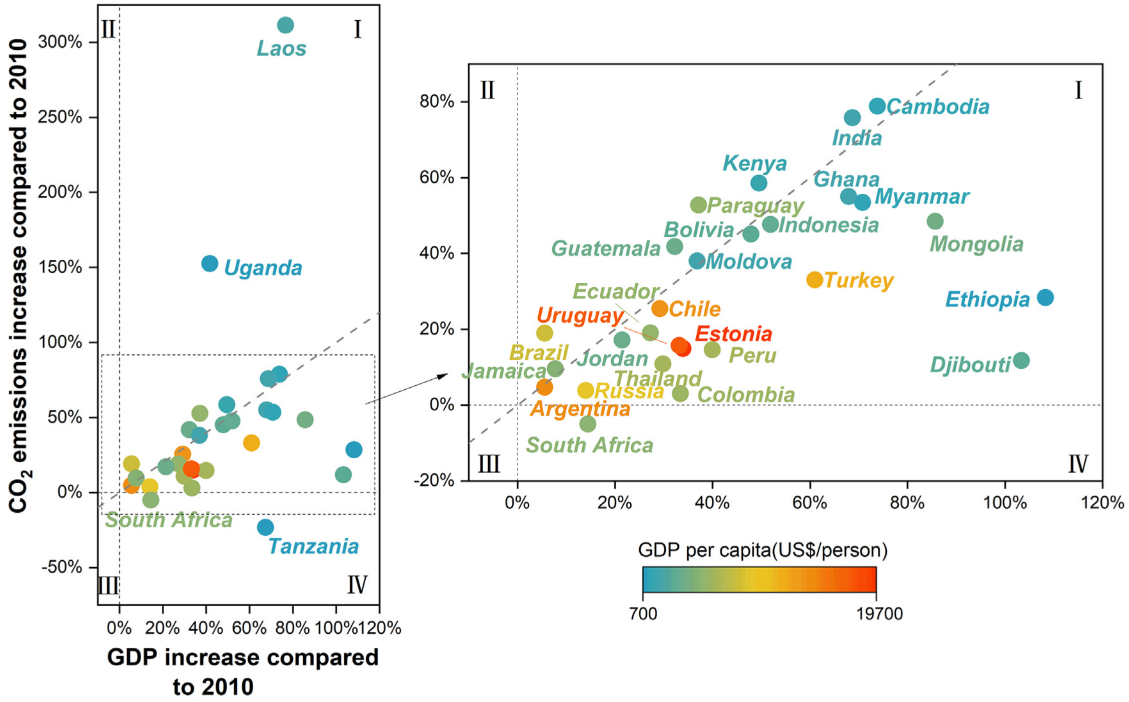

Figure 1. Overview of CO_2 emissions and economic development

The horizontal axis shows how much GDP in 2018 has increased compared with 2010, and the vertical axis shows how much CO_2 emissions in 2018 have increased compared with 2010. The dashed gray line signifies the point where the rate of change of emissions is equal to the rate of change of GDP. The colored circles represent the GDP per capita for each country in 2018.

33.9% in 2010–2018, and CO_2 emissions increased by 14.9%. In recent years, the emerging economies in group three have experienced a rapid growth in CO_2 emissions that has surpassed the GDP growth rate. Among these emerging economies, Kenya's CO_2 emissions in 2018 were about 1.6 times those in 2010, mainly due to rising emissions in the residential sector, where increasing use of biomass energy such as firewood for cooking has been noted. The Kenyan government introduced a zero value-added tax (VAT) for liquefied petroleum gas to reduce biomass consumption in 2016, but the subsidy was withdrawn in 2021, leading to increased reliance on biomass for cooking in low-income communities.[35] Meanwhile, Kenya's GDP grew by 49.5%. In addition, CO_2 emissions have also risen significantly in Laos, Uganda, India. and Cambodia.

Carbon intensity (CO_2 emissions per unit of GDP) is a quantitative way of emission reduction that combines the level of economic development with carbon emissions. Carbon intensity varies hugely across emerging economies; among the 30 countries studied, it ranges between 0.14 and 3.43 kg CO_2/$ GDP. Of these, the emerging economies in Africa had relatively high carbon intensity, while the figures for those in Latin America and the Caribbean were relatively low (Figure S1). For example, in Uruguay and Argentina in 2018, the carbon intensities were approximately 0.16 and 0.25 kg CO_2/$ GDP, respectively. This can be partly attributed to the relatively large proportion of value-added of tertiary industry in their industrial structures. By contrast, although the carbon intensity of emerging economies such as Ethiopia was decreasing year by year, it was still as high as 2.11 kg CO_2/$ GDP in 2018. That might be down to the increasing value-added of the manufacturing and construction industry brought about by the industrialization process and the use of related emission-intensive energy and traditional biomass for cooking (Figure S1 and Table S1).

The heterogeneity and time-series dynamic changes of energy structure

Across the emerging economies studied, there was significant variation in energy use over time. In some emerging economies of Africa and Asia, coal accounted for a relatively large proportion of the energy structure: for instance, 58.5% in South Africa and 58.9% in India in 2018 (Figure 2). Coal use in some emerging economies in these regions, including Cambodia, Laos, and Ethiopia, has increased significantly (Figure 2). In Laos, the amount of coal used in 2017 was 11.8 times that used in 2014, and the share of coal in the energy mix increased from 6.7% in 2014 to 40.6% in 2017 (Figure 2), in tandem with rapid growth in electricity generation. Likewise, the use of coal in Cambodia and Ethiopia in 2018 was some 44.2 and 13 times that in 2010, respectively. For Myanmar, though its share of oil soared from 29.1% in 2010 to 48.5% in 2017 (Figure 2), biomass still accounted for over 30% of total energy use. Similarly, the use of biomass in Kenya in 2018 was 1.5 times that used in 2010, a trend closely related to the large proportion of agricultural value added (Table S1). It is worth noting that in emerging economies such as Ethiopia, the proportion of agricultural value added exceeded that in the manufacturing and construction industry, suggesting that there is a lot of room for agricultural sector improvements in the future (Table S1). Because the majority of food

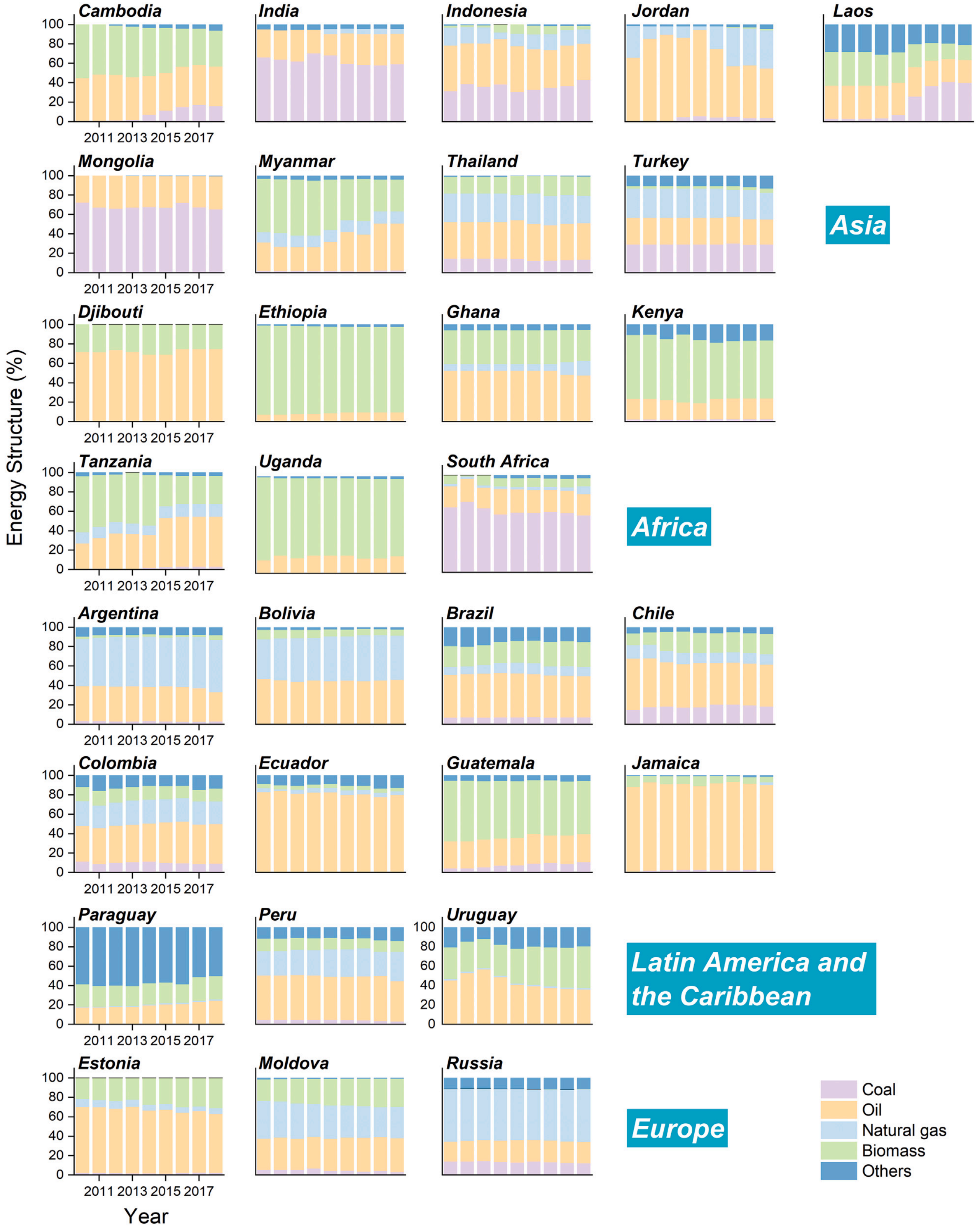

Figure 2. The changes of energy structure in 30 selective emerging economies, 2010–2018

The bars show the proportion in consumption about coal (purple), oil (orange), gas (light blue), biomass (green), and others (blue).

production operations are now concentrated in emerging economies, decarbonizing the sector and reducing biomass use are critical to lowering emissions. In this context, the sustainable use of biomass energy in emerging economies needs to be given more consideration. Turkey's GDP per capita in 2018, meanwhile, was quite high, topping $10,000. Its energy structure was fairly homogeneous, with coal, oil, and natural gas accounting for more than 25% of its total energy consumption.

In contrast to some emerging economies in Asia and Africa, those in Latin America and the Caribbean mainly rely on oil rather than coal as a source of energy. In Argentina, Bolivia, Peru, and Colombia, the prime energy sources are oil and gas (Figure 2); in Brazil, Chile, Guatemala, Uruguay, and Jamaica, oil and biomass energy predominate; and Ecuador depends primarily on oil and renewable energy. In terms of energy growth, the use of biomass in Argentina was more than 2-fold in 2018 compared with 2010. Bolivians increasingly turned to oil, natural gas, and renewable energy sources in addition to biomass. In Uruguay, the use of gas increased significantly (about 1.4 times in 2018 based on 2010). Meanwhile, most emerging economies with slow growth in the manufacturing and construction industry (an average annual growth rate of lower than 5% or even negative growth) and the highest proportion of service value added were located in Latin America and the Caribbean (Table S1).

Sectoral heterogeneity of CO_2 emissions

There is significant variation in levels of emissions at the region and sector levels due to differences in the use of coal. In 2018, some emerging economies, such as India, recorded high CO_2 emissions as a result of massive coal combustion (1,687.9 Mt) (Figure S2). In most emerging economies, the CO_2 emissions from coal combustion were emitted primarily by the electricity and heat sector (Figure 3A). For example, in Chile and Cambodia, coal-driven CO_2 emissions in the electricity and heating sector were as high as 96.1% and 91.5%, respectively. In Myanmar and Colombia, the nonmetal products sector was also found to be a source of coal-related emissions. CO_2 emissions from coal combustion in Brazil, Kenya, and Moldova were more diverse in sectoral distribution. Emissions from coal in Brazil were mainly spread across the sectors of electricity and heating (21.8%), logging and food (14.6%), machinery (13.2%), energy extraction (12.1%), minerals mining (11.8%), and metal products (11%). The sectors producing most coal-driven emissions in other countries included residential sector in Moldova (45%) and minerals mining in Kenya (76.3%). It is worth mentioning in this context that most emerging economies are at the forefront of manufacturing and production processes to meet the demands of the developed world's growing tertiary sector.

CO_2 emissions associated with the use of oil were mainly concentrated in the transportation sector in emerging economies (yellow area in Figure 3B), while the electricity and heating sector accounted for a relatively high proportion in Estonia. Moreover, the residential sector in Indonesia accounted for 34.7% of emissions from oil use. Intuitively, there is a diversification in terms of sectoral distribution of emissions from the use of natural gas in emerging economies, including Indonesia, India, and Turkey (Figure 3C). CO_2 emissions in Indonesia from natural gas were mainly distributed in the energy extraction and the electricity and heating sectors, while emissions in India were mainly concentrated in the transportation and the electricity and heating sectors. Natural gas-driven emissions in Turkey were mainly from the electricity and heating and residential sectors, accounting for 41.3% and 26.7%, respectively.

Emissions from the residential use of biomass in Guatemala and Colombia accounted for 96.9% and 88.3% of total biomass-driven emissions, respectively (Figure 3D). The level of traditional biomass use associated with the residential sector, which thus accounts for significant CO_2 emissions, indicates that there is considerable room for increasing biomass efficiency in these emerging economies. Apart from those associated with residential use (mainly for cooking), emissions from biomass were also driven by sectors including electricity and heating in other emerging economies. Argentina, for instance, mainly used biomass to generate power (21.2%, or 0.6 Mt; Figures 3D and S2).

Drivers of changes in CO_2 emissions

The study analyzes the breakdown, via factors of CO_2 emissions changes, of fossil fuels in the residential and industrial sectors (Figures 4 and S3). The drivers include emission coefficient (CO_2 emissions per unit energy); energy structure (the share of coal, oil, and gas); population; energy intensity (energy use per unit GDP, only in the industry); industrial structure (the share of primary, secondary, and tertiary, only in the industry); GDP per capita (only in the industry); and energy per capita (only in the residential sector). With the exception of South Africa, the factors that led to a decrease in CO_2 emissions from fossil fuels in most emerging economies from 2010 to 2018 did not ultimately counteract the causes contributing to rising CO_2 emissions. Thus, emissions have risen across emerging economies but to varying extents. In general, a rise in GDP per capita and population were the primary drivers of CO_2 emissions. Energy intensity, meanwhile, contributed to decreasing CO_2 emissions in the majority of emerging economies, whereas energy structure, industrial structure, and emission coefficient had different effects across countries, driving CO_2 emissions up or down.

The impact of energy structure on emissions differed among emerging economies. Lower coal and oil emissions were primarily responsible for this drop in emissions in Peru and Argentina, and increasing gas has contributed to a rise in CO_2 emissions. While their lower oil use lowered overall emissions, that impact was lessened by coal use, which simultaneously raised CO_2 emissions, particularly in Asia and including Cambodia and Laos. The case of Brazil helps in grasping how energy structure influences variations in CO_2 emissions. Here, oil was responsible for a 13.9 Mt (3.9%) reduction in CO_2 emissions. Coal and gas, on the other hand, have contributed to a rise in CO_2 emissions of 6.1 (1.7%) and 7.8 Mt (2.2%), respectively. In Jordan, the most important factor in reducing CO_2 emissions from 2010 to 2018 was reducing the share of oil in the energy mix, which resulted in a drop of 4.4 Mt (21.7%). The country's growth in CO_2 emissions, on the other hand, was highly dependent on its gas contribution.

With other factors remained constant, the overall industrial structure, including primary, secondary, and tertiary, in emerging economies had a variable impact on CO_2 emissions (Figures 4 and S3). The changes in industrial structure in emerging economies were associated with a decline in such emissions, for

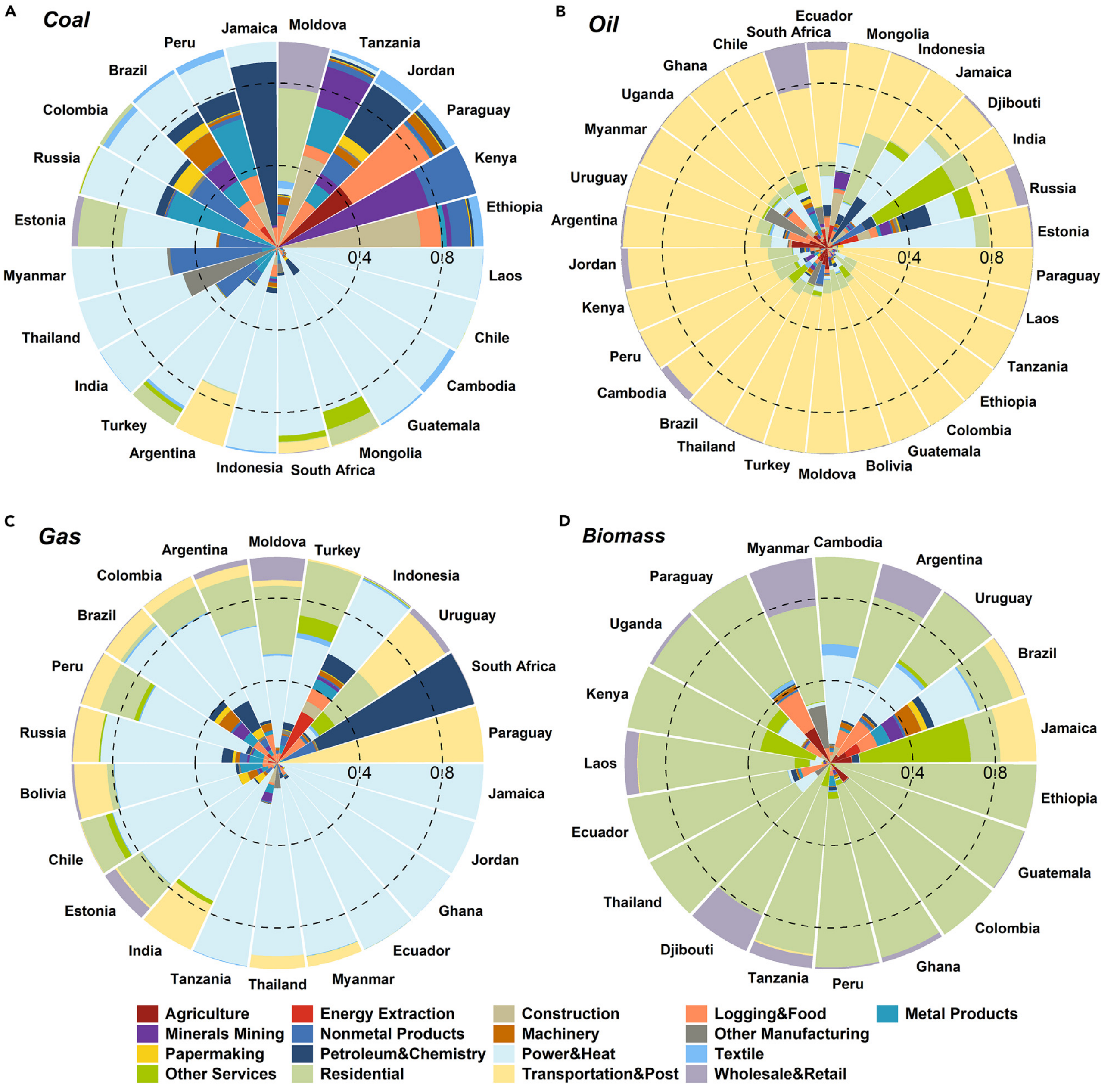

Figure 3. Distribution of CO_2 emissions caused by different types of energy use among sectors in 2018

Two circles demonstrate 40% and 80% of the emissions generated by energy use.

(A) The proportion of CO_2 emissions from coal combustion is shown to be distributed among 17 sectors.

(B–D) The proportion of CO_2 emissions from oil, gas, and biomass combustion, respectively, is shown as distributed among 17 sectors.

instance in Asia (India, Indonesia, Jordan, and Thailand); Africa (South Africa); Latin America and the Caribbean (Argentina, Bolivia, Brazil, Chile, Colombia, Ecuador, Guatemala, Peru, and Uruguay); and Europe (Moldova). Different industries have had various impacts on CO_2 emissions across emerging economies. Some emerging economies were clearly undergoing industrial structural transformation in the period studied. Manufacturing and construction triggered a drop in CO_2 emissions, whereas the service industry had the opposite effect. For instance, in Brazil, the increase in CO_2 emissions came from the share of service (4.5 Mt) (Figure 4). Meanwhile, manufacturing and construction contributed 18.2 Mt (5.1%) to the decrease in CO_2 emissions in Brazil. In Thailand, service—as a pillar of the industry, especially tourism—was associated with a rise in CO_2 emissions of 9.7 Mt (4.4%) from 2010 to 2018, while the shares of agriculture and of manufacturing and construction reduced CO_2 emissions by 1.5 and 16.8 Mt, respectively. Yet in Mongolia, the share of manufacturing and construction boosted CO_2 emissions by 0.6 Mt (5.4%), while the share of service triggered a reduction in CO_2 emissions of

CellPress
OPEN ACCESS

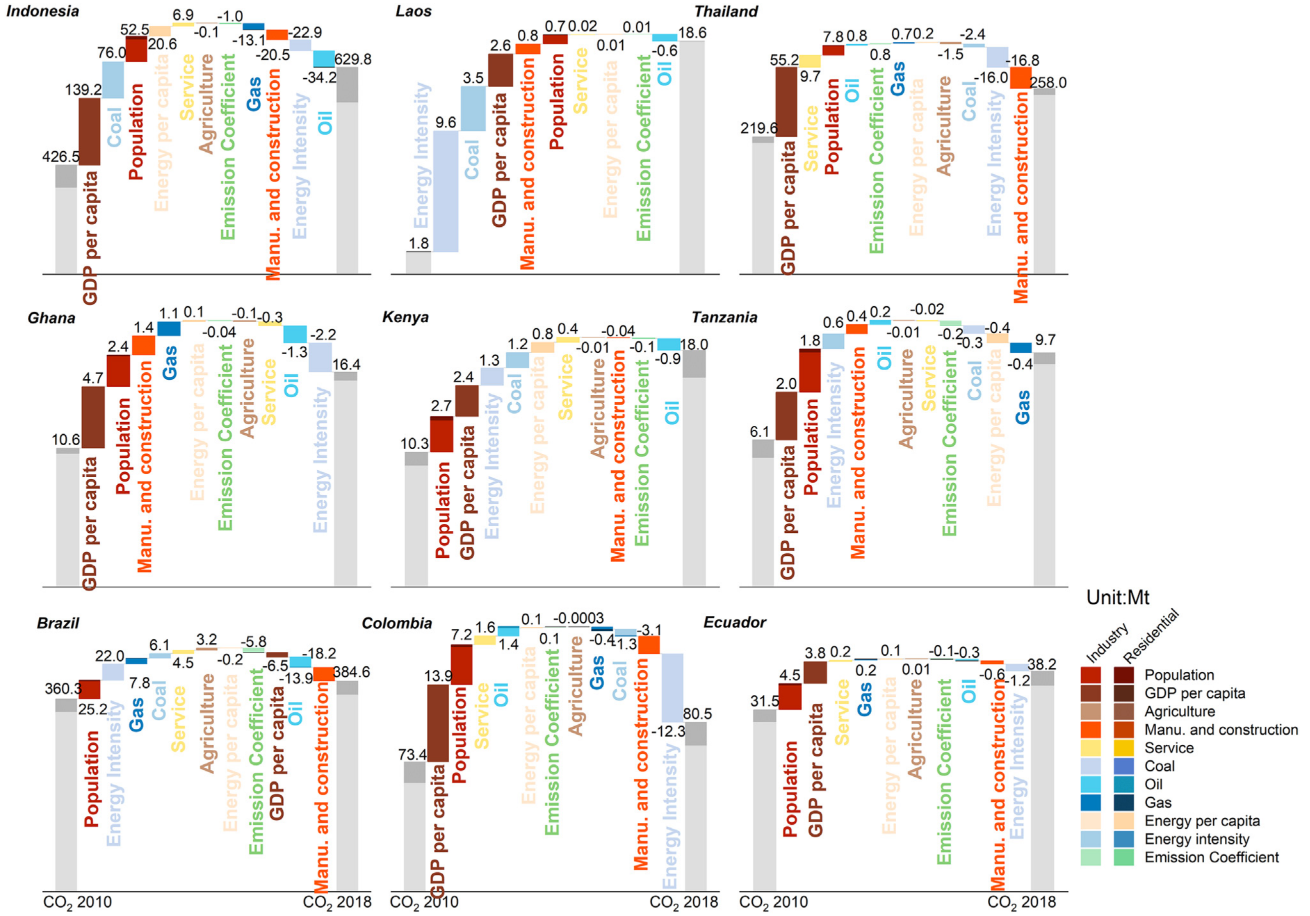

Figure 4. Drivers of CO_2 emissions changes about fossil fuels, 2010–2018

The driving factors of CO_2 emissions in the residential sector and industry are distinguished by differently colored bars and headings. Among them, the common drivers of the residential and industrial sectors are population; the share of coal, oil, and gas; and emission coefficient (CO_2 emissions per unit energy). Energy per capita is the driving factor in the residential sector, whereas GDP per capita, energy intensity (energy use per unit GDP), and the share of the primary (agriculture), secondary (manufacturing and construction), and tertiary (service) industries are driving factors in the industrial sector.

0.2 Mt (1.6%). When decomposition analysis includes biomass emissions, the outcomes are presented in Figure S4. Lowering biomass emissions was the main factor behind the decrease in emissions observed in Tanzania, and similar shifts in biomass emissions contributed to CO_2 emission reduction in other economies, especially in Myanmar and Laos. Elsewhere, particularly in Kenya, biomass use has contributed to a rise in CO_2 emissions.

DISCUSSION

Studies have exposed a gap between existing national policies and actions of climate change and meeting the Paris Agreement goals—that is, a bottleneck in achieving a temperature rise of no more than 2°C or 1.5°C.[36,37] Policies to curb emissions vary across countries. The formulation of net-zero targets has created a possibility for the realization of the Paris Agreement,[38] with 21 of the 30 selective emerging economies setting up corresponding net-zero targets (Table S1). Barring Turkey, Thailand, Indonesia, Russia, Ghana, and India, the remaining 15 of the 21 countries have set targets for carbon neutrality for 2050. Turkey and Thailand have established carbon neutrality objectives for 2053 and 2065, respectively. Ghana and India aim to achieve their net-zero emissions by 2070, with India for the first time committing to meet 50% of its energy requirements from renewable sources by 2030. Russia and Indonesia have set a 2060 target to achieve net-zero emissions. Despite the challenges, countries are now advancing their carbon neutrality targets in the form of renewable energy substitution; the usage of carbon capture, utilization, and storage (CCUS); and tapping ocean and terrestrial carbon sinks.[39,40]

Detailed emission inventories by energy types and sectors are important for the development of emission policies in emerging economies. Therefore, in order to better address climate change and global warming, we have collected energy data from 30 selective emerging economies through data crowdsourcing to complete the construction of emissions and thus analyze potential emission reduction pathways in scientific ways. In certain countries, such as South Africa and Tanzania, the relationship between economic development and CO_2 emissions has shifted in the other direction, toward the decline of emissions and the increase of economy—although whether this is a long-term, stable

trend is unknown. In the vast majority of emerging economies, economic development and emissions were still in a high-speed growth trajectory.

Numerous developed countries that are well known to the public have largely reached the emission peaks, whereas most developing countries have not and are still in the upward stage.[41] Also, the heterogeneity of emission patterns and drivers between countries has been demonstrated in our study and previous papers.[42,43] On the one hand, this study shows that energy intensity is the important driver for the decline of emissions in most emerging economies, especially Djibouti. By contrast, the energy intensity of some emerging economies even drives emission growth, such as in Kenya and Laos. On the other hand, the decline of energy intensity—that is, the improvement of energy efficiency—in numerous emerging economies cannot offset the increase in emissions caused by economic development and population. Thus, there is still room for optimizing energy intensity and carbon intensity to mitigate emissions.

Moreover, the adjustment of industrial structure in emerging economies has gradually become a key factor in low-carbon pathways. One of the plausible pathways for emerging economies to reduce emissions is therefore to lean toward the tertiary sectors. However, manufacturing and construction is the mainstay of emerging economies; thus, the industrial structure of these emerging economies cannot be excessively tilted to the tertiary sectors. Reduced consumption and changes in lifestyle choices will be required in developed nations to lower demand for secondary industries (manufacturing and construction) in emerging economies. Another way for the adjustment of industrial structure for decreasing emissions is to carry out internal adjustments in a specific industry, and especially manufacturing and construction, to speed up the transition to low-carbon industries such as high-end manufacturing. Adding technologies such as CCUS can increase industrial added value as well as reduce emissions.

In addition, adjusting the energy mix—through a gradual transition to renewable energy in the form of wind, solar, and hydro—is particularly important in regard to the secondary industry. The adjustment of the energy mix has long shown the decreasing effect of emissions in known developed countries, and the importance of renewable energy has also been estimated.[44] For example, Le Quéré et al. found that the largest contribution to the reduction of emissions, about 36%–73%, came from reductions in the share of fossil fuels over the period 2005–2015 in 18 developed countries.[45] Nevertheless, the energy transition in emerging economies is inadequate yet. The share of coal in Asia, such as in Laos and Cambodia, was still on the rise, which was one of the key factors contributing to the growth of emissions. For Tanzania and Myanmar, the declining share of biomass made the energy mix act as the driver for the reduction of emissions. Moreover, in India and South Africa, for instance, coal and oil dominated energy usage. Our study also showed that electricity and heat constituted the main source of CO_2 emissions in most emerging economies, indicating that these emerging economies will take some time for emissions to peak and that they still have a long way to go in reducing emissions associated with the electricity sector. Thus, it is key to identify the reasons for the growth of emissions in emerging economies, especially by focusing on the substitution of energy use within the secondary industry (manufacturing and construction).[46]

Emerging economies may face investment challenges in the future. Under the guidance and support of policies, emerging economies need to actively attract foreign investment and adjust the flow of funds to the field of renewable energy. Meanwhile, there is substantial potential for promoting low-carbon technology transfer[47] from developed countries to emerging economies to improve energy efficiency and reduce carbon intensity. COP26 brought to the forefront the divide between developed and developing nations, as the latter seek financial support to decouple economic development and emissions through renewable technologies. Given that emerging economies' per-capita emissions are low from a climate justice perspective, there is a case to be made for developed nations to provide financial assistance and technology transfer to enable these economies to meet productive energy use goals.

Among sectors in Africa, the residential sector is responsible for most emissions from biomass combustion and also uses fossil fuels such as gas and coal. Thus, to tackle emission reduction in this sector in emerging economies, tools such as policies are needed to reduce the use of traditional biomass energy and fossil fuels and ease the adoption of electricity as much as possible.[48–50] Decarbonization can be achieved through the displacement of traditional biomass such as deforestation for household cooking and fossil fuels by electrification. Meanwhile, the comparison of emission patterns and reduction factors at regional and national scales is more worthy of consideration. Latin America and the Caribbean have ample renewable energy, such as hydro and solar, which has been better utilized in recent years.[51] Africa, with similar latitude, is also rich in renewable energy, but there is limited utilization. Therefore, the development experiences of renewable energy (considering opportunities for renewables investments and increasing the share of renewable energy while meeting the rapidly growing energy demand) in Latin America and the Caribbean,[52] such as Brazil, Uruguay, and Paraguay, can be used to further explore renewable energy in Africa. Especially, renewable energy such as solar and hydro in Africa hereby optimize the energy structure and play a deserved role in reducing emissions. At the detailed country level, we still clearly see that coal increases Indonesia's emissions and its use in electricity production. The key to reducing emissions lies in the more rational use of energy in all sectors, especially in electricity production to encourage the use of renewable energy such as geothermal energy (holding about 40% of the world[53]). Indonesia is an island country (with many islands), which makes economic development and energy distribution uneven. It is necessary to consider the energy distribution according to national circumstances and try to promote energy transformation by developing transport and the power grid.

In aggregate terms, accurate and refined data on emissions for emerging economies are the basis for the deployment of emission reduction strategies. Through data science, the effectiveness and causes of emission reduction are demonstrated more comprehensively. These 30 emerging economies selected for this study are representative to some extent. They include not only large emerging economies such as India and Brazil but also small and island emerging economies such as Jamaica. However, due to different geographical locations and resource endowments, the emission patterns of each emerging economy are heterogeneous, and potential energy transformation is

inconsistent. The methodology used in this article is in accordance with the Intergovernmental Panel on Climate Change (IPCC) guidelines and is consistent with databases such as the IEA and the Global Carbon Budget (GCB). This article only considers energy-related emissions and does not account for industrial process emissions. Therefore, energy consumption in activity data and emission factors are the main sources of uncertainty. In particular, emission factors differ among emerging economies, and the availability of official emission factor information is limited. The accounting of energy-related emissions includes unsustainable biomass emissions, which is also one of the sources of uncertainty. In addition, the refinement of sectoral matching indicators will further improve the sector's accounting results. We will continue to track the emission accounting and emission reduction potential of emerging economies in the future. More efforts are needed to conduct research and evaluate emerging economies.

Methods

Carbon accounting

The compilation of emissions in emerging economies includes emissions related to energy use but excludes emissions from industrial processes. Accounting of CO_2 emissions for emerging economies according to IPCC guidelines is shown in Equation 1:

$$CE = \sum_i \sum_j CE_{ij} = \sum_i \sum_j AD_{ij} \times EF_j, \quad \text{(Equation 1)}$$

where i stands for industry and j represents the energy type including coal, oil, gas, and biomass. CE_{ij} is the carbon emissions of different industries and energy varieties. Given that our inventories are about emissions related to energy use, AD_{ij} is energy consumption data. EF is the emission factor, which can measure emissions by energy use per unit. Among them, in some emerging economies, the use of biomass is mainly sourced from wood and charcoal through deforestation, which is unsustainable over a certain period. Thus, the use of biomass in some emerging economies generates corresponding CO_2 emissions in the process of accounting.

$$EF_j = NCV_j \times CC_j \times O_j \quad \text{(Equation 2)}$$

The EF is obtained by multiplying the net calorific value (NCV), the carbon content (CC), and the oxidation rate (O), where NCV is the heat released by per unit of energy use, and the CC is the emissions per unit of heat. In addition, we also unify sectors of emerging economies according to some data or indicators, such as data of energy consumption or economic indicators. For a more comprehensive understanding, please refer to our published work, where we have presented detailed information.[54]

Index decomposition analysis

Index decomposition analysis (IDA) and structural decomposition analysis (SDA) have been used extensively to analyze the contributions of economic and social factors to changes of greenhouse gas and air pollutants in recent years.[55] SDA needs to be deployed in combination with the input-output table, which reflects the interrelation among various sectors across countries in a certain period.[56,57] Usually, the release time of the input-output table by institutions is discontinuous, which has a time lag. In contrast, IDA has relatively low data requirements and is relatively flexible on the selection of the time period studied. Moreover, IDA includes two other index methods, the Laspeyres[58,59] and the Divisia.[60] Given the characteristics of complete decomposition, convincing results, and theoretical basis, the accumulation method in the logarithmic mean division index (LMDI) is preferred in this study. We have used the LMDI method[61,62] to analyze the changes in CO_2 emissions of emerging economies. In this study, we denote CO_2 emissions of emerging economies as follows:

$$\begin{aligned} C &= \sum_{i=1}^{3} \sum_{j=1}^{3} C_{ij} = \sum_{i=1}^{3} \sum_{j=1}^{3} \frac{C_{ij}}{E_{ij}} \times \frac{E_{ij}}{E_i} \times \frac{E_i}{G_i} \times \frac{G_i}{G} \times \frac{G}{P} \times P \\ &= \sum_{i=1}^{3} \sum_{j=1}^{3} CI_{ij} \times ES_{ij} \times EI_{ij} \times S_i \times Y \times P \end{aligned} \quad \text{(Equation 3)}$$

This equation quantifies the total CO_2 emissions from human sources as the product of several factors, building a bridge between emissions and important elements such as energy, economy, and population, where i represents industry, which is divided into the primary, secondary, and tertiary industries, and j represents the type of energy, which is divided into coal, oil, and gas. We decompose CO_2 emissions into CI_{ij}, ES_{ij}, EI_{ij}, S_i, Y, and P. CI_{ij} represents carbon intensity (C_{ij}/E_{ij}), which refers to the amount of CO_2 emissions per unit of energy. ES_{ij} represents the energy structure (E_{ij}/E_i), which describes the proportion of coal, oil, natural gas, biomass, and others in total energy. EI_{ij} represents the energy intensity (E_i/G_i), which refers to the amount of energy required per unit of GDP. S_i is the industrial structure (G_i/G), that is, the share of primary, secondary, and tertiary in total. Y is GDP per capita (G/P). P is population.

Specifically, we separately decompose CO_2 emissions of the residential sector into the following factors:

$$\begin{aligned} C^r &= \sum_{j=1}^{3} C_j^r = \sum_{j=1}^{3} \frac{C_j^r}{E_j^r} \times \frac{E_j^r}{E^r} \times \frac{E^r}{P} \times P \\ &= \sum_{j=1}^{3} CI_j^r \times ES_j^r \times EP^r \times P \end{aligned} \quad \text{(Equation 4)}$$

Similarly, in Equation 4, C^r denotes carbon emissions in the residential sector. CI_j^r represents the carbon intensity (C_j^r/E_j^r). ES_j^r represents the energy structure (E_j^r/E^r). EP^r represents energy per capita (E^r/P). P is population.

Thus, changes in CO_2 emissions are decomposed into the following six factors over the specific time period.

$$\begin{aligned} \Delta C &= C_t - C_0 \\ &= \Delta C_{CI} + \Delta C_{ES} + \Delta C_{EI} + \Delta C_S \\ &\quad + \Delta C_Y + \Delta C_P \end{aligned} \quad \text{(Equation 5)}$$

The contribution of each part in Equation 5 can be expressed as

$$\Delta C_{CI} = \sum_{i=1}^{3} \sum_{j=1}^{3} \frac{C_{ij}^t - C_{ij}^0}{\ln\left(C_{ij}^t\right) - \ln\left(C_{ij}^0\right)} \times \ln\left(\frac{CI_{ij}^t}{CI_{ij}^0}\right), \quad \text{(Equation 6)}$$

$$\Delta C_{ES} = \sum_{i=1}^{3}\sum_{j=1}^{3}\frac{C_{ij}^{t} - C_{ij}^{0}}{\ln\left(C_{ij}^{t}\right) - \ln\left(C_{ij}^{0}\right)} \times \ln\left(\frac{ES_{ij}^{t}}{ES_{ij}^{0}}\right), \quad \text{(Equation 7)}$$

$$\Delta C_{EI} = \sum_{i=1}^{3}\sum_{j=1}^{3}\frac{C_{ij}^{t} - C_{ij}^{0}}{\ln\left(C_{ij}^{t}\right) - \ln\left(C_{ij}^{0}\right)} \times \ln\left(\frac{EI_{ij}^{t}}{EI_{ij}^{0}}\right), \quad \text{(Equation 8)}$$

$$\Delta C_{S} = \sum_{i=1}^{3}\sum_{j=1}^{3}\frac{C_{ij}^{t} - C_{ij}^{0}}{\ln\left(C_{ij}^{t}\right) - \ln\left(C_{ij}^{0}\right)} \times \ln\left(\frac{S_{i}^{t}}{S_{i}^{0}}\right), \quad \text{(Equation 9)}$$

$$\Delta C_{Y} = \sum_{i=1}^{3}\sum_{j=1}^{3}\frac{C_{ij}^{t} - C_{ij}^{0}}{\ln\left(C_{ij}^{t}\right) - \ln\left(C_{ij}^{0}\right)} \times \ln\left(\frac{Y^{t}}{Y^{0}}\right), \quad \text{(Equation 10)}$$

$$\Delta C_{P} = \sum_{i=1}^{3}\sum_{j=1}^{3}\frac{C_{ij}^{t} - C_{ij}^{0}}{\ln\left(C_{ij}^{t}\right) - \ln\left(C_{ij}^{0}\right)} \times \ln\left(\frac{P^{t}}{P^{0}}\right). \quad \text{(Equation 11)}$$

Similarly, CO_2 emissions in the residential sector of emerging economies are analyzed by the LMDI method:

$$\begin{aligned}\Delta C &= C_t - C_0 \\ &= \Delta C_{CI} + \Delta C_{ES} + \Delta C_{EP} + \Delta C_P\end{aligned} \quad \text{(Equation 12)}$$

When accounting for biomass emissions in the decomposition analysis, the energy mix encompasses a range of sources, including coal, oil, gas, biomass, and others.

EXPERIMENTAL PROCEDURES

Resource availability

Lead contact

Further information should be directed to and will be fulfilled by the lead contact, Dabo Guan (guandabo@mail.tsinghua.edu.cn).

Materials availability

This study did not generate new unique materials.

Data and code availability

This study employs the energy data from the region's or nation's own statistics (Table S3) for CO_2 emissions accounting. GDP and population data are from the United Nations,[63] and GDP data are shown in US dollars (at constant 2015 prices). The data on CO_2 emissions that support the findings of this study and the code of driving forces behind emissions change in this study can be seen from Zenodo[64] (https://doi.org/10.5281/zenodo.7871178).

SUPPLEMENTAL INFORMATION

Supplemental information can be found online at https://doi.org/10.1016/j.patter.2023.100760.

ACKNOWLEDGMENTS

We acknowledge support from the National Key R&D Program of China (2022YFE0208700), the National Natural Science Foundation of China (72140001 and 7221101088), the UK Natural Environment Research Council (NE/V002414/1), and the Royal Society (IEC/NSFC/191520). Meanwhile, we appreciate members of our group, Yida Sun, Chang Tan, Binyuan Liu, Song Qing, Qi Hao, Huaxi Peng, Qi Huang, Jinglei Wang, Huiting Wu, and Lin Xiao, for help with data collection and compilation.

AUTHOR CONTRIBUTIONS

J.M. and D.G. led the construction of the project. S.L. calculated the results of the study. S.L., C.C., and J.M. mainly prepared the manuscript. All authors participated in the writing of the manuscript. J.M. coordinated the whole study. J.M., Y.L., Y.S., W.Z., P.P., J.Y., and D.G. checked the manuscript.

DECLARATION OF INTERESTS

The authors declare no competing interests.

Received: October 12, 2022
Revised: December 30, 2022
Accepted: May 2, 2023
Published: May 30, 2023

REFERENCES

1. Levin, K., and Rich, D. (2017). Turning points: trends in countries' reaching peak greenhouse gas emissions over time. World Resour. Inst. https://www.wri.org/research/turning-points-trends-countries-reaching-peak-greenhouse-gas-emissions-over-time.
2. Hultman, N.E., Clarke, L., Frisch, C., Kennedy, K., McJeon, H., Cyrs, T., Hansel, P., Bodnar, P., Manion, M., Edwards, M.R., et al. (2020). Fusing subnational with national climate action is central to decarbonization: the case of the United States. Nat. Commun. *11*, 5255. https://doi.org/10.1038/s41467-020-18903-w.
3. Victoria, M., Zhu, K., Brown, T., Andresen, G.B., and Greiner, M. (2020). Early decarbonisation of the European energy system pays off. Nat. Commun. *11*, 6223. https://doi.org/10.1038/s41467-020-20015-4.
4. Peters, G.P., Le Quéré, C., Andrew, R.M., Canadell, J.G., Friedlingstein, P., Ilyina, T., Jackson, R.B., Joos, F., Korsbakken, J.I., McKinley, G.A., et al. (2017). Towards real-time verification of CO2 emissions. Nat. Clim. Chang. *7*, 848–850. https://doi.org/10.1038/s41558-017-0013-9.
5. Lei, R., Feng, S., and Lauvaux, T. (2020). Country-scale trends in air pollution and fossil fuel CO2 emissions during 2001–2018: confronting the roles of national policies and economic growth. Environ. Res. Lett. *16,* 014006. https://doi.org/10.1088/1748-9326/abc9e1.
6. Meng, J., Mi, Z., Guan, D., Li, J., Tao, S., Li, Y., Feng, K., Liu, J., Liu, Z., Wang, X., et al. (2018). The rise of South–South trade and its effect on global CO2 emissions. Nat. Commun. *9*, 1871. https://doi.org/10.1038/s41467-018-04337-y.
7. Ravindranath, N.H., and Sathaye, J.A. (2002). Climate change and developing countries. In Climate Change and Developing Countries, N.H. Ravindranath and J.A. Sathaye, eds. (Springer Netherlands), pp. 247–265. https://doi.org/10.1007/0-306-47980-X_9.
8. Gore, T., Ghosh, E., Nazareth, A., Kartha, S., and Dabi, N. (2021). Carbon inequality in 2030: per capita consumption emissions and the 1.5 ºC goal. https://oxfamilibrary.openrepository.com/bitstream/handle/10546/621305/bn-carbon-inequality-2030-051121-en.pdf?sequence=1.
9. Ayompe, L.M., Davis, S.J., and Egoh, B.N. (2020). Trends and drivers of African fossil fuel CO2 emissions 1990–2017. Environ. Res. Lett. *15*. 124039. https://doi.org/10.1088/1748-9326/abc64f.
10. Li, S., Meng, J., Zheng, H., Zhang, N., Huo, J., Li, Y., and Guan, D. (2021). The driving forces behind the change in energy consumption in developing countries. Environ. Res. Lett. *16*. 054002. https://doi.org/10.1088/1748-9326/abde05.
11. Cronin, J., Hughes, N., Tomei, J., Caiado Couto, L., Ali, M., Kizilcec, V., Adewole, A., Bisaga, I., Broad, O., Parikh, P., et al. (2021). Embedding justice in the 1.5°C transition: a transdisciplinary research agenda. Renew. Sustain. Energy Transit. *1*. 100001. https://doi.org/10.1016/j.rset.2021.100001.
12. Raupach, M.R., Marland, G., Ciais, P., Le Quéré, C., Canadell, J.G., Klepper, G., and Field, C.B. (2007). Global and regional drivers of accelerating CO2 emissions. Proc. Natl. Acad. Sci. USA *104*, 10288–10293. https://doi.org/10.1073/pnas.0700609104.

CellPress
OPEN ACCESS

13. Steckel, J.C., Edenhofer, O., and Jakob, M. (2015). Drivers for the renaissance of coal. Proc. Natl. Acad. Sci. USA *112*. E3775. E3781. https://doi.org/10.1073/pnas.1422722112.

14. Zheng, X., Lu, Y., Yuan, J., Baninla, Y., Zhang, S., Stenseth, N.C., Hessen, D.O., Tian, H., Obersteiner, M., and Chen, D. (2020). Drivers of change in China's energy-related CO2 emissions. Proc. Natl. Acad. Sci. USA *117*, 29–36. https://doi.org/10.1073/pnas.1908513117.

15. Guan, D., Meng, J., Reiner, D.M., Zhang, N., Shan, Y., Mi, Z., Shao, S., Liu, Z., Zhang, Q., and Davis, S.J. (2018). Structural decline in China's CO2 emissions through transitions in industry and energy systems. Nat. Geosci. *11*, 551–555. https://doi.org/10.1038/s41561-018-0161-1.

16. Wang, Q., Cao, C., Guo, R., Li, X., Lu, L., Wang, W., and Li, S. (2016). Drivers for energy consumption: a comparative analysis of China and India. Renew. Sustain. Energy Rev. *40*, 954–961. https://doi.org/10.1016/j.rser.2016.04.048.

17. Pachauri, S. (2014). Household electricity access a trivial contributor to CO2 emissions growth in India. Nat. Clim. Chang. *4*, 1073–1076. https://doi.org/10.1038/nclimate2414.

18. Huang, Q., Zheng, H., Li, J., Meng, J., Liu, Y., Wang, Z., Zhang, N., Li, Y., and Guan, D. (2021). Heterogeneity of consumption-based carbon emissions and driving forces in Indian states. Adv. Appl. Energy *4*. 100039. https://doi.org/10.1016/j.adapen.2021.100039.

19. Chong, C.H., Tan, W.X., Ting, Z.J., Liu, P., Ma, L., Li, Z., and Ni, W. (2019). The driving factors of energy-related CO2 emission growth in Malaysia: the LMDI decomposition method based on energy allocation analysis. Renew. Sustain. Energy Rev. *115*. 109356. https://doi.org/10.1016/j.rser.2019.109356.

20. İpek Tunç, G., Türüt-Aşık, S., and Akbostancı, E. (2009). A decomposition analysis of CO2 emissions from energy use: Turkish case. Energy Pol. *37*, 4689–4699. https://doi.org/10.1016/j.enpol.2009.06.019.

21. Taka, G.N., Huong, T.T., Shah, I.H., and Park, H.-S. (2020). Determinants of energy-based CO2 emissions in Ethiopia: a decomposition analysis from 1990 to 2017. Sustainability *12*, 4175. https://doi.org/10.3390/su12104175.

22. Steckel, J.C., Hilaire, J., Jakob, M., and Edenhofer, O. (2020). Coal and carbonization in sub-Saharan Africa. Nat. Clim. Chang. *10*, 83–88. https://doi.org/10.1038/s41558-019-0649-8.

23. Bistline, J.E.T., and Blanford, G.J. (2021). Impact of carbon dioxide removal technologies on deep decarbonization of the electric power sector. Nat. Commun. *12*, 3732. https://doi.org/10.1038/s41467-021-23554-6.

24. Liao, C., Wang, S., Zhang, Y., Song, D., and Zhang, C. (2019). Driving forces and clustering analysis of provincial-level CO2 emissions from the power sector in China from 2005 to 2015. J. Clean. Prod. *240,* 118026. https://doi.org/10.1016/j.jclepro.2019.118026.

25. Shen, G., Ru, M., Du, W., Zhu, X., Zhong, Q., Chen, Y., Shen, H., Yun, X., Meng, W., Liu, J., et al. (2019). Impacts of air pollutants from rural Chinese households under the rapid residential energy transition. Nat. Commun. *10*, 3405. https://doi.org/10.1038/s41467-019-11453-w.

26. Goldstein, B., Gounaridis, D., and Newell, J.P. (2020). The carbon footprint of household energy use in the United States. Proc. Natl. Acad. Sci. USA *117*, 19122–19130. https://doi.org/10.1073/pnas.1922205117.

27. Berrill, P., Gillingham, K.T., and Hertwich, E.G. (2021). Linking housing policy, housing typology, and residential energy demand in the United States. Environ. Sci. Technol. *55*, 2224–2233. https://doi.org/10.1021/acs.est.0c05696.

28. Maraseni, T.N., Qu, J., and Zeng, J. (2015). A comparison of trends and magnitudes of household carbon emissions between China, Canada and UK. Environ. Dev. *15*, 103–119. https://doi.org/10.1016/j.envdev.2015.04.001.

29. Azevedo, V.G., Sartori, S., and Campos, L.M. (2018). CO2 emissions: a quantitative analysis among the BRICS nations. Renew. Sustain. Energy Rev. *81*, 107–115. https://doi.org/10.1016/j.rser.2017.07.027.

30. International Energy Agency (2023). International Energy Agency Greenhouse Gas Emissions from Energy, 1751-2021, 13th Edition. https://doi.org/10.5257/iea/co2/2021.

31. World Bank. Population growth (annual %). https://data.worldbank.org/indicator/SP.POP.GROW.

32. Ouedraogo, N.S. (2017). Africa energy future: alternative scenarios and their implications for sustainable development strategies. Energy Pol. *106*, 457–471. https://doi.org/10.1016/j.enpol.2017.03.021.

33. International Energy Agency (2020). World energy outlook 2020. https://www.iea.org/reports/world-energy-outlook-2020.

34. Puranasamriddhi, A., and Parikh, P. (2021). Off-grid energy and economic prosperity. https://www.ucl.ac.uk/bartlett/construction/sites/bartlett_construction/files/00impact_of_off-grid_in_ssa_report_final.pdf.

35. Shupler, M., Pope, D., Puzzolo, E., Menya, D., Mwitari, J., Muchiri, E., Perros, T., Parikh, P., Lokolile, L., and Wandera, F. (2022). COP26 and SDG7 goals under threat: 16% VAT on LPG reverses progress made in clean cooking adoption in Kenya. https://doi.org/10.13140/RG.2.2.15161.85603.

36. Geiges, A., Nauels, A., Parra, P.Y., Andrijevic, M., Hare, W., Pfleiderer, P., Schaeffer, M., and Schleussner, C.-F. (2020). Incremental improvements of 2030 targets insufficient to achieve the Paris Agreement goals. Earth Syst. Dyn. *11*, 697–708. https://doi.org/10.5194/esd-11-697-2020.

37. Roelfsema, M., van Soest, H.L., Harmsen, M., van Vuuren, D.P., Bertram, C., den Elzen, M., Höhne, N., Iacobuta, G., Krey, V., Kriegler, E., et al. (2020). Taking stock of national climate policies to evaluate implementation of the Paris Agreement. Nat. Commun. *11*, 2096. https://doi.org/10.1038/s41467-020-15414-6.

38. Höhne, N., Gidden, M.J., den Elzen, M., Hans, F., Fyson, C., Geiges, A., Jeffery, M.L., Gonzales-Zuñiga, S., Mooldijk, S., Hare, W., and Rogelj, J. (2021). Wave of net zero emission targets opens window to meeting the Paris Agreement. Nat. Clim. Chang. *11*, 820–822. https://doi.org/10.1038/s41558-021-01142-2.

39. Wang, F., Harindintwali, J.D., Yuan, Z., Wang, M., Wang, F., Li, S., Yin, Z., Huang, L., Fu, Y., Li, L., et al. (2021). Technologies and perspectives for achieving carbon neutrality. Innovation *2,* 100180. https://doi.org/10.1016/j.xinn.2021.100180.

40. Liu, Z., Deng, Z., He, G., Wang, H., Zhang, X., Lin, J., Qi, Y., and Liang, X. (2021). Challenges and opportunities for carbon neutrality in China. Nat. Rev. Earth Environ. *3*, 141–155. https://doi.org/10.1038/s43017-021-00244-x.

41. Xia, Q., Wang, H., Liu, X., and Pan, X. (2021). Drivers of global and national CO2 emissions changes 2000–2017. Clim. Pol. *21*, 604–615. https://doi.org/10.1080/14693062.2020.1864267.

42. Shahiduzzaman, M., Layton, A., and Alam, K. (2015). Decomposition of energy-related CO2 emissions in Australia: challenges and policy implications. Econ. Anal. Pol. *45*, 100–111. https://doi.org/10.1016/j.eap.2014.12.001.

43. Wang, Z., Jiang, Q., Dong, K., Mubarik, M.S., and Dong, X. (2020). Decomposition of the US CO2 emissions and its mitigation potential: an aggregate and sectoral analysis. Energy Pol. *147,* 111925. https://doi.org/10.1016/j.enpol.2020.111925.

44. Moutinho, V., Madaleno, M., Inglesi-Lotz, R., and Dogan, E. (2018). Factors affecting CO2 emissions in top countries on renewable energies: a LMDI decomposition application. Renew. Sustain. Energy Rev. *90*, 605–622. https://doi.org/10.1016/j.rser.2018.02.009.

45. Le Quéré, C., Korsbakken, J.I., Wilson, C., Tosun, J., Andrew, R., Andres, R.J., Canadell, J.G., Jordan, A., Peters, G.P., and van Vuuren, D.P. (2019). Drivers of declining CO2 emissions in 18 developed economies. Nat. Clim. Chang. *9*, 213–217. https://doi.org/10.1038/s41558-019-0419-7.

46. Semieniuk, G., Taylor, L., Rezai, A., and Foley, D.K. (2021). Plausible energy demand patterns in a growing global economy with climate policy. Nat. Clim. Chang. *11*, 313–318. https://doi.org/10.1038/s41558-020-00975-7.

47. Goldthau, A., Eicke, L., and Weko, S. (2020). The global energy transition and the global South. In The Geopolitics of the Global Energy Transition, M. Hafner and S. Tagliapietra, eds. (Springer International Publishing), pp. 319–339. https://doi.org/10.1007/978-3-030-39066-2_14.

48. Kaygusuz, K. (2012). Energy for sustainable development: a case of developing countries. Renew. Sustain. Energy Rev. *16*, 1116–1126. https://doi.org/10.1016/j.rser.2011.11.013.

49. Nock, D., Levin, T., and Baker, E. (2020). Changing the policy paradigm: a benefit maximization approach to electricity planning in developing countries. Appl. Energy *264*, 114583. https://doi.org/10.1016/j.apenergy.2020.114583.

50. Cummins, M., and Gillanders, R. (2020). Greasing the turbines? Corruption and access to electricity in Africa. Energy Pol. *137*, 111188. https://doi.org/10.1016/j.enpol.2019.111188.

51. Koengkan, M. (2018). The decline of environmental degradation by renewable energy consumption in the MERCOSUR countries: an approach with ARDL modeling. Environ. Syst. Decis. *38*, 415–425. https://doi.org/10.1007/s10669-018-9671-z.

52. Kieffer, G., and López-Peña, Á. (2016). Renewable energy market analysis: Latin America (International Renewable Energy Agency). https://www.irena.org/publications/2016/Nov/Renewable-Energy-Market-Analysis-Latin-America.

53. Yudha, S., Tjahjono, B., and Longhurst, P. (2022). Unearthing the dynamics of Indonesia's geothermal energy development. Energies *15*, 5009. https://doi.org/10.3390/en15145009.

54. Cui, C., Li, S., Zhao, W., Liu, B., Shan, Y., and Guan, D. (2023). Energy-related CO2 emission accounts and datasets for 40 emerging economies in 2010–2019. Earth Syst. Sci. Data *15*, 1317–1328. https://doi.org/10.5194/essd-15-1317-2023.

55. Hoekstra, R., and van den Bergh, J.C. (2003). Comparing structural and index decomposition analysis. Energy Econ. *25*, 39–64. https://doi.org/10.1016/s0140-9883(02)00059-2.

56. Meng, J., Yang, H., Yi, K., Liu, J., Guan, D., Liu, Z., Mi, Z., Coffman, D.M., Wang, X., Zhong, Q., et al. (2019). The slowdown in global air-pollutant emission growth and driving factors. One Earth *1*, 138–148. https://doi.org/10.1016/j.oneear.2019.08.013.

57. Zheng, J., Ding, W., Li, B., Yang, Y., Guan, D., and Wang, S. (2019). The slowdown in China's carbon emissions growth in the new phase of economic development. One Earth *694*, 240–241. https://doi.org/10.1016/j.oneear.2019.

58. Ang, B.W., Huang, H.C., and Mu, A.R. (2009). Properties and linkages of some index decomposition analysis methods. Energy Pol. *37*, 4624–4632. https://doi.org/10.1016/j.enpol.2009.06.017.

59. Peters, G.P., Andrew, R.M., Canadell, J.G., Fuss, S., Jackson, R.B., Korsbakken, J., Le Quéré, C., and Nakicenovic, N. (2017). Key indicators to track current progress and future ambition of the Paris Agreement. Nat. Clim. Chang. *7*, 118–122. https://doi.org/10.1038/nclimate3202.

60. Ang, B.W., and Choi, K.-H. (1997). Decomposition of aggregate energy and gas emission intensities for industry: a refined Divisia index method. Energy J. *18*. https://doi.org/10.5547/ISSN0195-6574-EJ-Vol18-No3-3.

61. Shan, Y., Fang, S., Cai, B., Zhou, Y., Li, D., Feng, K., and Hubacek, K. (2021). Chinese cities exhibit varying degrees of decoupling of economic growth and CO_2 emissions between 2005 and 2015. One Earth *4*, 124–134. https://doi.org/10.1016/j.oneear.2020.12.004.

62. Ang, B.W. (2005). The LMDI approach to decomposition analysis: a practical guide. Energy Pol. *33*, 867–871. https://doi.org/10.1016/j.enpol.2003.

63. United Nations Statistics Division (2022). UNdata: a world of information. http://data.un.org/Default.aspx.

64. Li, S., Cui, C., Meng, J., Li, Y., Shan, Y., Zhao, W., Parikh, P., Yao, J., and Guan, D. (2023). The heterogeneous driving forces behind carbon emissions change in 30 selective emerging economies (Zenodo). https://doi.org/10.5281/zenodo.7871178.

Patterns, Volume *4*

Supplemental information

The heterogeneous driving forces behind carbon emissions change in 30 selective emerging economies

Shuping Li, Can Cui, Jing Meng, Yuan Li, Yuli Shan, Weichen Zhao, Priti Parikh, Jiawei Yao, and Dabo Guan

Supplemental Figures

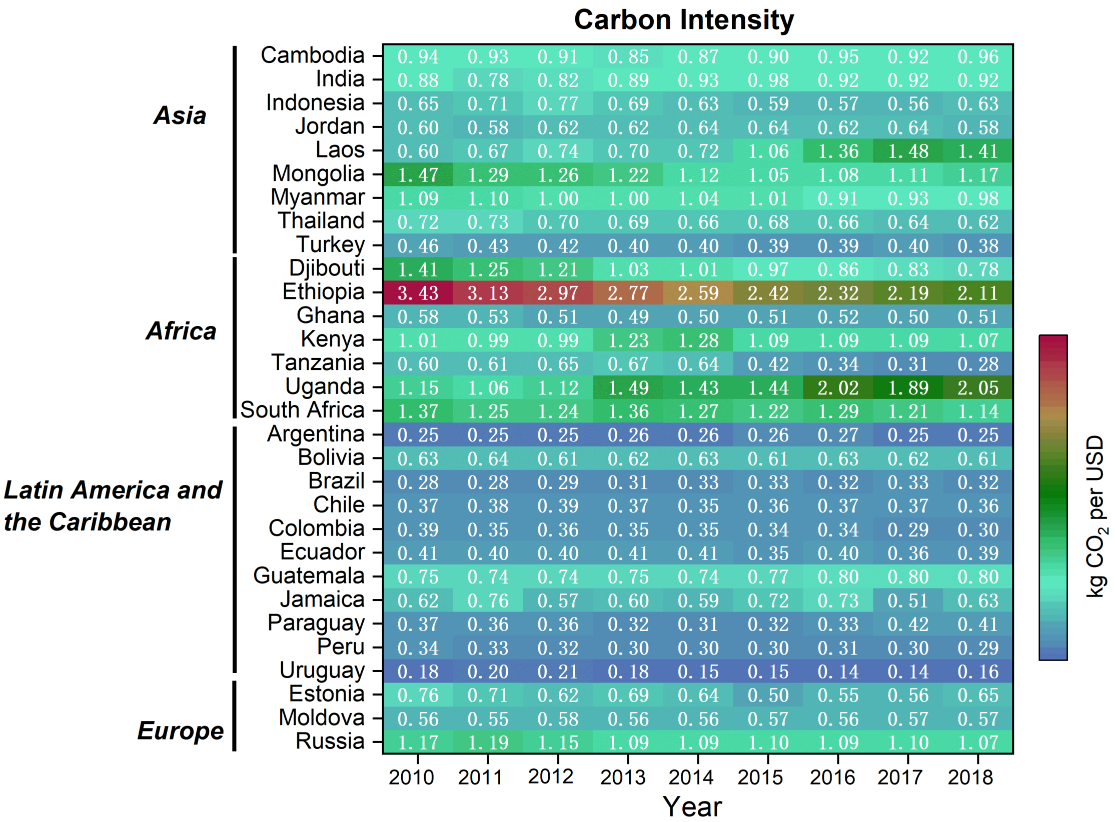

Figure S1. Carbon intensity (CO_2 emissions per GDP) in 30 selective emerging economies, 2010-2018.

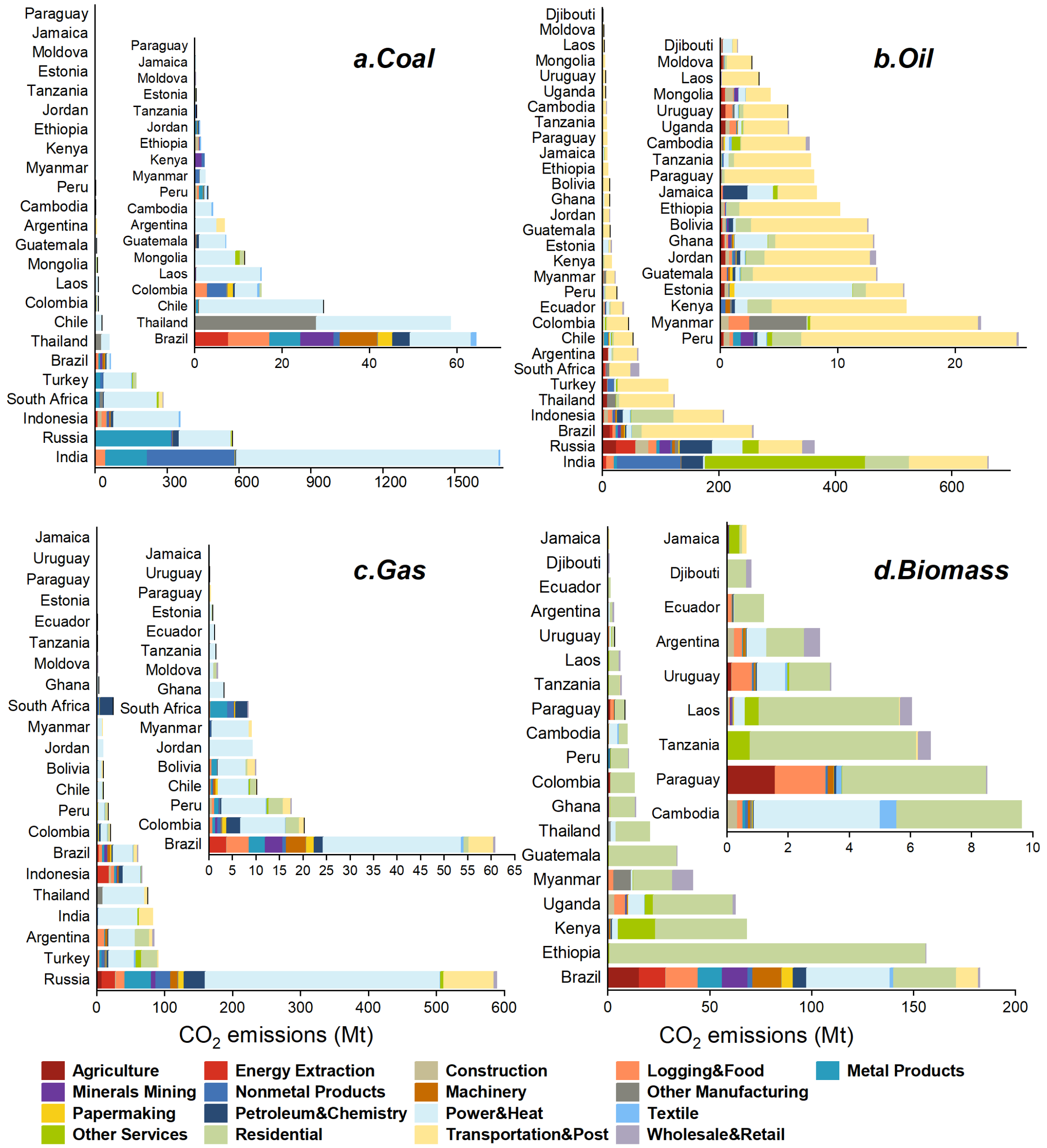

Figure S2. CO_2 emissions caused by different types of energy use, 2018.

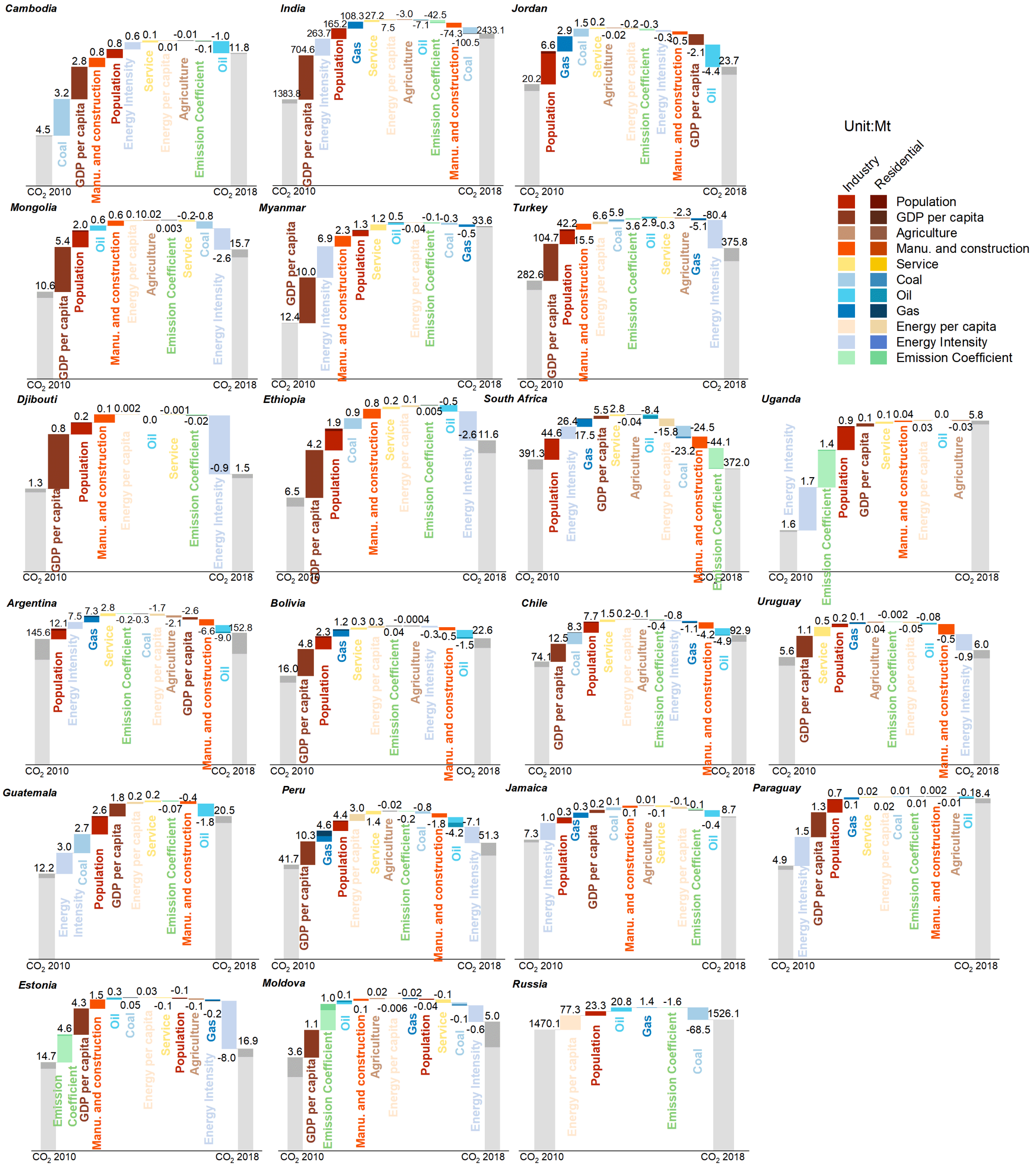

Figure S3. Drivers of CO_2 emissions changes about fossil fuels, 2010-2018.

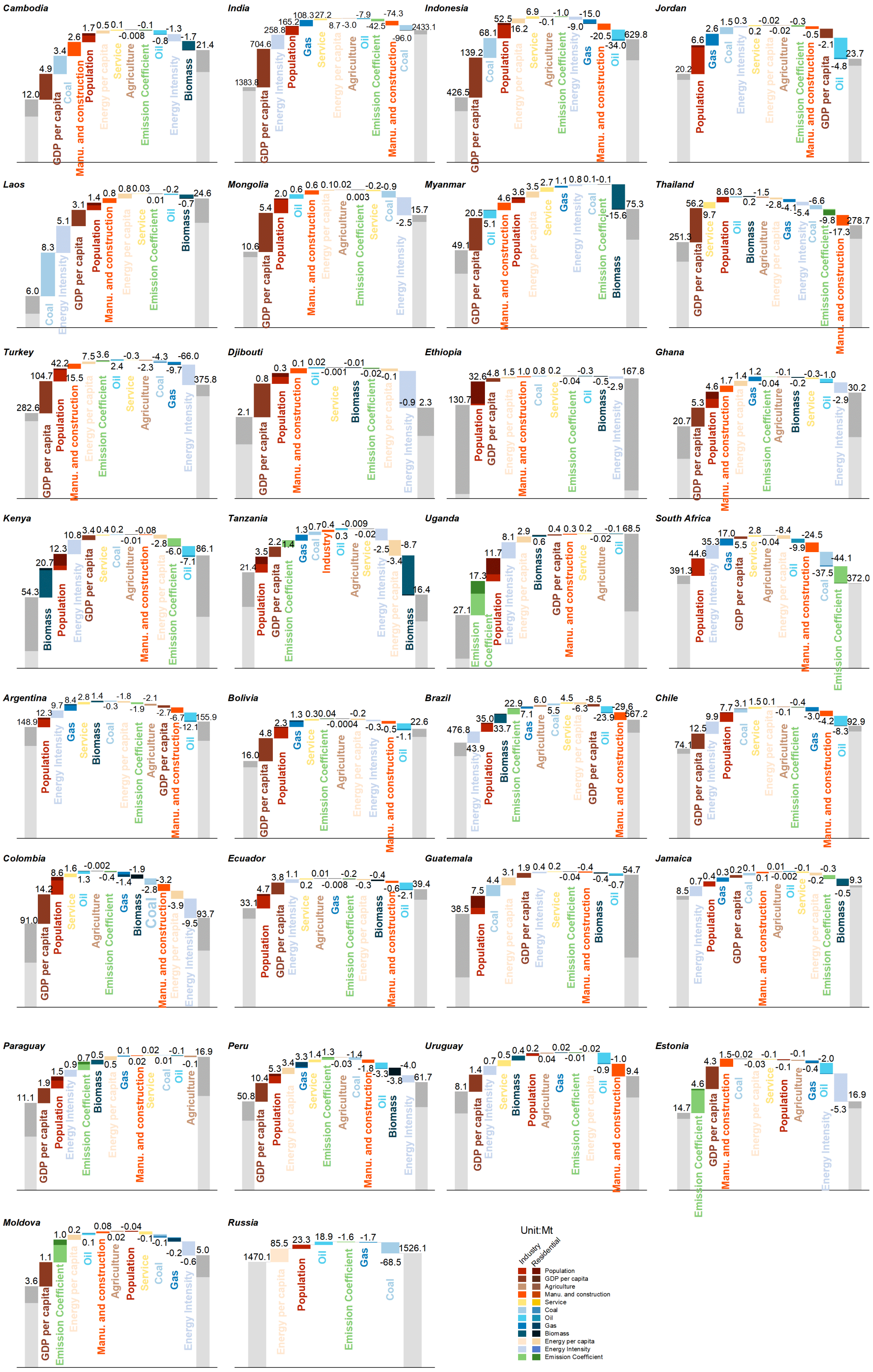

Figure S4. Drivers of CO_2 emissions changes including fossil fuels and biomass, 2010-2018. Among them, due to the availability of Russian, the industrial structure has not been analyzed. The decomposition factors in Russia only include emission coefficient, energy mix, energy per capita and population.

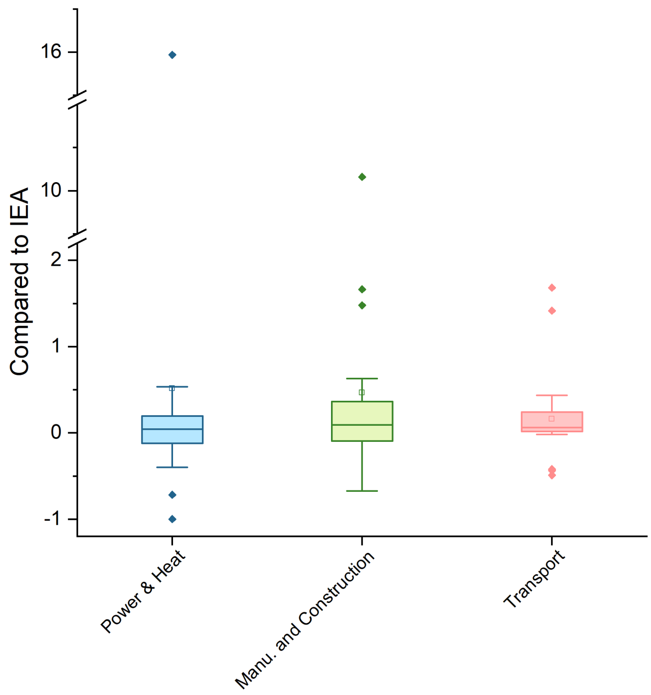

Figure S5. Data comparison with IEA about fossil fuels emissions among sectors in 2018.

Supplemental Tables

Table S1. Average annual growth rate of the secondary industry (manufacturing and construction) during 2010-2018 in 30 selective emerging economies, industrial structure in 2018, and net zero target.

	Country	Average annual growth rate of the manufacturing and construction	Agriculture	Manufacturing and construction	Service	Ranking	Net-zero target
The share of agriculture > The share of manufacturing and construction	Ethiopia	20.64%	34.15%	22.66%	43.18%	III>I>II	Yes
Average annual growth rate of manufacturing and construction < 5%	Uruguay	-1.93%	7.47%	20.24%	72.29%	III>II>I	Yes
	Brazil	-0.88%	5.51%	21.59%	72.90%	III>II>I	Yes
	Argentina	-0.51%	5.20%	26.16%	68.63%	III>II>I	Yes
	South Africa	0.63%	2.36%	28.36%	69.28%	III>II>I	Yes
	Jamaica	1.10%	7.84%	22.55%	69.61%	III>II>I	Yes
	Thailand	1.84%	8.64%	34.56%	56.81%	III>II>I	Yes
	Russia	1.84%	4.30%	33.77%	61.93%	III>II>I	Yes
	Jordan	2.04%	5.15%	27.38%	67.47%	III>II>I	No
	Chile	2.05%	4.02%	31.59%	64.39%	III>II>I	Yes
	Colombia	2.50%	6.84%	29.62%	63.54%	III>II>I	Yes
	Ecuador	2.60%	10.69%	32.75%	56.56%	III>II>I	Yes
	Guatemala	2.87%	10.56%	23.15%	66.29%	III>II>I	No
	Peru	3.15%	7.97%	32.66%	59.36%	III>II>I	Yes
	Bolivia	4.24%	13.03%	29.42%	57.55%	III>II>I	No
	Paraguay	4.28%	9.97%	37.30%	52.73%	III>II>I	No
	Indonesia	4.48%	13.51%	40.58%	45.91%	III>II>I	Yes
	Moldova	4.71%	15.23%	25.78%	58.99%	III>II>I	No
	Kenya	4.97%	19.17%	19.21%	61.62%	III>II>I	No
	Uganda	4.58%	22.77%	29.99%	47.23%	III>II>I	No
Average annual growth rate of manufacturing and construction > 5%	Estonia	5.30%	1.94%	29.15%	68.91%	III>II>I	Yes
	India	5.75%	17.05%	29.65%	53.30%	III>II>I	Yes
	Turkey	7.32%	7.06%	31.53%	61.41%	III>II>I	Yes
	Mongolia	8.62%	14.73%	32.81%	52.46%	III>II>I	No
	Tanzania	9.28%	27.67%	29.27%	43.07%	III>II>I	Yes
	Myanmar	9.40%	22.29%	36.04%	41.67%	III>II>I	Yes
	Laos	9.69%	17.51%	34.78%	47.71%	III>II>I	Yes
	Ghana	10.94%	21.05%	38.17%	40.78%	III>II>I	Yes
	Djibouti	11.67%	1.52%	17.30%	81.18%	III>II>I	No
	Cambodia	11.94%	24.06%	33.70%	42.24%	III>II>I	Yes

Table S2 List of Sectoral classification.

No.	Sector_47	Sector_17
**1**	Agriculture, forestry, hunting, fishing and husbandry	Agriculture, forestry, hunting, fishing and husbandry
**2**	Coal Mining and Dressing	Coal Mining, Petroleum and Natural Gas Extraction
**3**	Petroleum and Natural Gas Extraction	
**4**	Ferrous Metals Mining and Dressing	Minerals Mining and Dressing
**5**	Nonferrous Metals Mining and Dressing	
**6**	Nonmetal Minerals Mining and Dressing	
**7**	Other Minerals Mining and Dressing	
**8**	Logging and Transport of Wood and Bamboo	Logging, Timber, Food, Beverage and Tobacco
**9**	Food Processing	
**10**	Food Production	
**11**	Beverage Production	
**12**	Tobacco Processing	
**13**	Timber Processing, Bamboo, Cane, Palm Fiber & Straw Products	
**14**	Furniture Manufacturing	
**15**	Textile Industry	Textile, Garments and Leather
**16**	Garments and Other Fiber Products	
**17**	Leather, Furs, Down and Related Products	
**18**	Papermaking and Paper Products	Papermaking, Printing and Cultural
**19**	Printing and Record Medium Reproduction	
**20**	Cultural, Educational and Sports Articles	
**21**	Petroleum Processing and Coking	Petroleum Processing, Raw Chemical, and Medical
**22**	Raw Chemical Materials and Chemical Products	
**23**	Medical and Pharmaceutical Products	
**24**	Chemical Fiber	
**25**	Rubber Products	Nonmetal Mineral Products
**26**	Plastic Products	
**27**	Nonmetal Mineral Products	
**28**	Smelting and Pressing of Ferrous Metals	Metal Smelting, Pressing and Products
**29**	Smelting and Pressing of Nonferrous Metals	
**30**	Metal Products	
**31**	Ordinary Machinery	Ordinary, Special, Transportation, Electric, Electronic and Instrument Machinery
**32**	Equipment for Special Purposes	
**33**	Transportation Equipment	
**34**	Electric Equipment and Machinery	
**35**	Electronic and Telecommunications	

	Equipment	
**36**	Instruments, Meters, Cultural and Office Machinery	
**37**	Other Manufacturing Industry	Other Manufacturing and Waste
**38**	Scrap and waste	
**39**	Production and Supply of Electric Power, Steam and Hot Water	Production of Electricity, Heat, Gas and Tap Water
**40**	Production and Supply of Gas	
**41**	Production and Supply of Tap Water	
**42**	Construction	Construction
**43**	Transportation, Storage, Post and Telecommunication Services	Transportation, Storage, Post and Telecommunication Services
**44**	Wholesale, Retail Trade and Catering Services	Wholesale, Retail Trade and Catering Services
**45**	Other Services	Other Services
**46**	Urban	Residential
**47**	Rural	

Data sources

Table S3 Data sources of energy.

Country	Website
Argentina	https://www.argentina.gob.ar/economia/energia/hidrocarburos/balances-energeticos
Bolivia	https://www.hidrocarburos.gob.bo/
Brazil	https://www.epe.gov.br/sites-pt/publicacoes-dados-abertos/publicacoes/PublicacoesArquivos/publicacao-377/topico-494/BEN%202019%20Completo%20WEB.pdf
Chile	http://energiaabierta.cl/visualizaciones/balance-de-energia/
Colombia	https://www1.upme.gov.co/InformacionCifras/Paginas/BECOCONSULTA.aspx
Djibouti	https://au-afrec.org/
Ecuador	https://www.recursosyenergia. gob.ec/
Estonia	http://pub.stat.ee/px-web.2001/I_Databas/Economy/07Energy/02Energy_consumption_and_production/01Annual_statistics/01Annual_statistics.asp
Ethiopia	http://www.csa.gov.et/
Ghana	http://www.energycom.gov.gh/files
Guatemala	https://mem.gob.gt/
Jordan	https://www.memr.gov.jo/Default/Ar
Kenya	https://unstats.un.org/unsd/energystats/pubs/balance/
Cambodia	https://www.eria.org/RPR_FY2015_No.8_Chapter_2.pdf
Laos	https://www.eria.org/publications/energy-demand-and-supply-of-the-lao-peoples-democratic-republic-2010-2018/
Indonesia	https://www.bps.go.id/

Moldova	https://statbank.statistica.md/PxWeb/pxweb/ro/40%20Statistica%20economica/40%20Statistica%20economica__15%20ENE__serii%20anuale/ENE020100.px/?rxid=b2ff27d7-0b96-43c9-934b-42e1a2a9a774
Myanmar	https://www.eria.org/publications/energy-demand-and-supply-of-the-republic-of-the-union-of-myanmar-2010-2017/
Mongolia	https://www.1212.mn/Stat.aspx?LIST_ID=976_L11&type=tables
Peru	https://sinia.minam.gob.pe/
Paraguay	http://www.dgeec.gov.py/
Thailand	https://www.dede.go.th/ewt_news.php?nid=47340
Turkey	https://www.dunyaenerji.org.tr/turkiye-enerji-denge-tablolari/
Tanzania	https://au-afrec.org/
Uganda	https://au-afrec.org/
Uruguay	https://www.ine.gub.uy/inicio
Jamaica	https://www.mset.gov.jm/document-category/energy-balances/
Russia	https://fedstat.ru/indicator
India	http://mospi.gov.in/
South Africa	http://www.energy.gov.za/files/media/Energy_Balances.html

Data comparison

Table S4. Data comparison with IEA about fossil fuels emissions. Unit: million tons.

Country	Year	IEA	CEADs
Argentina	2010	162.4	145.6
	2011	171.2	155.4
	2012	172.6	156.7
	2013	177.4	162.8
	2014	173.9	159.8
	2015	179.8	166.0
	2016	178.1	165.4
	2017	173.9	161.9
	2018	171.2	152.8
Bolivia	2010	13.6	16.0
	2011	14.8	17.0
	2012	15.8	17.0
	2013	16.8	18.5
	2014	18.1	19.8
	2015	18.5	20.0
	2016	19.8	21.5
	2017	20.2	22.1
	2018	20.3	22.6
Brazil	2010	373.2	360.3
	2011	392.2	378.5
	2012	425.5	410.5
	2013	455.6	439.2

	2014	479.7	453.6
	2015	455.9	431.6
	2016	420.1	395.9
	2017	430.0	406.6
	2018	408.1	384.6
Chile	2010	68.6	74.1
	2011	75.3	82.3
	2012	77.2	86.8
	2013	81.9	85.9
	2014	75.5	82.8
	2015	81.1	87.0
	2016	85.3	91.3
	2017	86.2	92.8
	2018	85.8	92.9
Colombia	2010	59.2	73.4
	2011	63.9	71.1
	2012	63.5	76.1
	2013	71.5	79.5
	2014	73.4	83.8
	2015	74.1	83.8
	2016	77.2	86.8
	2017	69.5	75.5
	2018	73.2	80.5
Djibouti	2010	0.5	1.3
	2011	0.5	1.3
	2012	0.5	1.5
	2013	0.5	1.4
	2014	0.3	1.4
	2015	0.4	1.4
	2016	0.3	1.5
	2017	0.3	1.5
	2018	0.3	1.5
Ecuador	2010	31.6	31.5
	2011	32.1	33.4
	2012	33.3	34.9
	2013	35.7	37.7
	2014	37.4	39.6
	2015	37.2	33.4
	2016	35.5	37.5
	2017	34.4	34.7
	2018	36.4	38.2
Estonia	2010	18.2	14.7
	2011	18.1	14.7

	2012	16.6	13.2
	2013	18.0	15.2
	2014	16.9	14.5
	2015	14.0	11.4
	2016	15.5	12.9
	2017	16.3	14.0
	2018	15.2	16.9
Ethiopia	2010	5.8	6.5
	2011	6.8	6.7
	2012	7.2	7.7
	2013	8.4	8.4
	2014	10.4	9.4
	2015	10.1	10.5
	2016	12.0	10.9
	2017	12.4	11.2
	2018	13.3	11.6
Ghana	2010	10.3	10.6
	2011	10.7	11.0
	2012	12.8	11.5
	2013	13.7	12.0
	2014	13.1	12.6
	2015	14.0	13.1
	2016	14.2	13.7
	2017	14.5	14.9
	2018	16.4	16.4
Guatemala	2010	10.4	12.2
	2011	10.7	12.5
	2012	11.1	13.1
	2013	11.9	14.3
	2014	12.6	15.1
	2015	15.0	18.3
	2016	16.1	19.7
	2017	15.2	19.0
	2018	16.9	20.5
Jordan	2010	18.8	20.2
	2011	19.8	20.0
	2012	22.7	22.1
	2013	22.3	22.6
	2014	24.0	24.2
	2015	23.7	24.5
	2016	23.1	24.4
	2017	24.4	25.9
	2018	23.0	23.7

Kenya	2010	11.6	10.3
	2011	11.7	10.7
	2012	10.7	11.1
	2013	11.9	12.0
	2014	13.8	13.6
	2015	14.9	16.0
	2016	16.0	16.7
	2017	17.0	17.3
	2018	16.2	18.0
Cambodia	2010	4.8	4.5
	2011	4.9	5.2
	2012	5.2	5.4
	2013	5.2	5.2
	2014	6.4	6.2
	2015	7.8	7.4
	2016	9.6	9.7
	2017	10.4	10.4
	2018	10.2	11.8
Laos	2010	2.4	1.8
	2011	2.6	2.1
	2012	2.7	2.5
	2013	3.3	2.8
	2014	3.5	3.6
	2015	7.7	9.2
	2016	14.4	14.9
	2017	17.6	18.3
	2018	17.8	18.6
Indonesia	2010	391.7	426.5
	2011	450.3	497.2
	2012	451.4	573.5
	2013	417.3	538.2
	2014	452.9	515.5
	2015	458.6	508.1
	2016	449.7	517.2
	2017	481.7	534.8
	2018	533.0	629.8
Moldova	2010	7.9	3.6
	2011	7.9	3.8
	2012	7.7	4.0
	2013	6.7	4.1
	2014	7.2	4.3
	2015	7.6	4.4
	2016	7.7	4.6

	2017	7.5	4.8
	2018	8.0	5.0
Myanmar	2010	7.9	12.4
	2011	8.5	13.2
	2012	11.6	12.0
	2013	13.2	12.9
	2014	16.5	17.3
	2015	18.7	22.6
	2016	20.9	21.2
	2017	30.4	29.7
	2018	30.3	33.6
Mongolia	2010	14.1	10.6
	2011	15.6	11.0
	2012	17.0	12.0
	2013	18.3	13.0
	2014	17.9	12.8
	2015	17.1	12.4
	2016	18.0	12.8
	2017	19.3	14.0
	2018	21.1	15.7
Peru	2010	41.5	41.7
	2011	44.9	42.7
	2012	44.1	45.0
	2013	45.2	45.6
	2014	48.1	46.9
	2015	49.5	48.3
	2016	52.2	52.5
	2017	49.8	50.8
	2018	50.1	51.3
Paraguay	2010	4.8	4.9
	2011	5.0	5.1
	2012	4.9	5.3
	2013	5.1	5.2
	2014	5.4	5.5
	2015	6.0	6.1
	2016	6.8	6.9
	2017	7.7	8.0
	2018	8.1	8.4
Thailand	2010	221.9	219.6
	2011	220.1	223.9
	2012	236.6	228.4
	2013	245.0	232.9
	2014	240.9	221.6

	2015	248.0	237.7
	2016	244.1	252.5
	2017	244.1	254.3
	2018	241.4	258.0
Turkey	2010	267.8	282.6
	2011	287.2	292.1
	2012	298.4	302.1
	2013	285.2	312.3
	2014	307.2	322.9
	2015	318.8	333.9
	2016	338.6	349.0
	2017	378.3	382.3
	2018	369.4	375.8
Tanzania	2010	6.0	6.1
	2011	7.1	7.8
	2012	8.6	9.9
	2013	9.8	10.2
	2014	9.0	10.2
	2015	9.6	11.4
	2016	9.1	10.4
	2017	10.0	10.1
	2018	10.3	9.7
Uganda	2010	3.0	1.6
	2011	3.3	3.3
	2012	3.3	2.8
	2013	3.3	3.8
	2014	3.7	4.1
	2015	4.4	4.4
	2016	4.6	4.7
	2017	4.8	4.8
	2018	5.5	5.8
Uruguay	2010	6.0	5.6
	2011	7.2	7.0
	2012	8.2	7.9
	2013	7.0	6.8
	2014	6.2	5.9
	2015	6.3	6.1
	2016	6.2	6.0
	2017	5.8	5.9
	2018	6.2	6.0
Jamaica	2010	7.2	7.3
	2011	7.2	9.6
	2012	6.6	7.1

	2013	7.0	7.4
	2014	6.8	7.2
	2015	6.7	9.2
	2016	7.2	9.8
	2017	6.9	6.9
	2018	8.3	8.7
Russia	2010	1505.3	1470.1
	2011	1580.4	1553.7
	2012	1583.1	1565.9
	2013	1543.3	1504.6
	2014	1525.8	1510.5
	2015	1504.6	1502.7
	2016	1478.5	1495.4
	2017	1506.3	1526.3
	2018	1566.7	1526.4
India	2010	1571.5	1383.8
	2011	1661.8	1284.6
	2012	1804.4	1434.8
	2013	1860.1	1638.2
	2014	2026.3	1846.1
	2015	2034.7	2098.4
	2016	2066.5	2134.2
	2017	2183.4	2290.7
	2018	2315.3	2433.1
South Africa	2010	420.3	391.3
	2011	404.7	366.6
	2012	422.3	373.2
	2013	432.3	418.8
	2014	443.3	397.8
	2015	419.6	386.9
	2016	419.9	410.4
	2017	429.7	390.1
	2018	434.1	372.0

Table S5. Data comparison with IEA about fossil fuels emissions by sectors in 2018. Unit: million tons.

	IEA	CEADs	IEA	CEADs	IEA	CEADs
	Power and Heat	Power and Heat	Manu. and Construction	Manu. and Construction	Transport	Transport
Argentina	47.07	48.73	20.72	19.72	47.79	47.13
Bolivia	4.00	6.13	2.25	3.20	10.71	11.49
Brazil	62.32	51.87	89.03	104.51	191.87	194.65
Chile	32.95	36.37	14.33	15.77	28.33	32.32

Colombia	13.01	16.81	15.72	19.31	29.34	37.16
Djibouti	0.05	0.80	0.02	0.26	0.14	0.37
Ecuador	5.78	6.40	3.04	7.53	19.16	20.17
Estonia	11.43	10.77	0.66	1.1	2.46	3.17
Ethiopia	0.00	0.00	4.89	1.99	6.84	8.50
Ghana	5.19	5.95	1.94	1.14	8.06	8.31
Guatemala	5.27	6.36	1.94	2.65	8.56	10.52
Jordan	9.02	9.66	1.62	2.55	9.01	8.98
Kenya	1.46	1.04	2.99	3.42	9.67	11.41
Cambodia	3.42	4.14	0.78	1.05	5.12	5.50
Laos	14.04	14.76	0.63	0.65	3.12	3.11
Indonesia	217.00	311.53	112.07	157.10	150.99	84.99
Moldova	3.26	0.92	1.08	0.35	2.10	2.16
Myanmar	7.61	9.09	8.48	9.42	6.10	14.73
Mongolia	13.55	9.86	1.96	1.58	2.17	2.13
Peru	10.94	10.75	8.12	8.50	24.18	25.30
Paraguay	0.00	0.00	0.22	0.13	7.61	7.97
Thailand	87.39	92.57	48.17	52.12	72.11	98.18
Turkey	144.22	156.42	72.69	72.53	83.37	86.16
Tanzania	2.74	1.65	1.94	1.11	5.06	6.45
Uganda	0.23	0.30	1.17	1.10	3.39	3.73
Uruguay	0.32	0.32	0.91	0.89	3.63	3.81
Jamaica	2.79	2.45	2.82	2.55	2.25	3.23
Russia	815.80	615.12	250.55	667.36	258.86	149.85
India	1197.16	1155.07	565.29	767.76	305.33	155.06
South Africa	233.28	220.68	54.24	73.03	55.60	50.92

CO_2 emissions about fossil fuels different from IEA for interquartile ranges covering 50% of the data ranging from -12.2%-19.4% for electricity and heat production, 9.4%-36.1% for manu. industries and construction, and 1.5%-24.3% for transport (Figure S5 and Table S5). The outliers observed in the sectors of Power & Heat and Manu. and Construction in Figure S5 can be attributed to different CO_2 emission for Djibouti. To quantify CO_2 emissions of Djibouti, the energy data used in this study were primarily sourced from the energy balance tables published on the African Energy Commission (AFREC). In contrast, the information of energy balance for Djibouti is not published in the IEA. The CO_2 emissions of Djibouti published in the Greenhouse Gas Emissions from Energy database by the IEA are estimated using data from the United Nations Statistics Division (UNSD) 2017 Energy Balances publication.

In addition, the IEA provides emissions of other and biofuels and renewable wastes. Other emissions include industrial waste and non-renewable municipal waste emissions. Biofuels and renewable wastes include biofuels (primary solid biofuels, biogases, biogasoline, biodiesels, bio jet kerosene and other liquid biofuels) and renewable wastes. However, biofuels and renewable wastes emissions are not counted in the total emissions. Due to the different ways of consideration, we divide biomass into

sustainable and unsustainable. For sustainable biomass we do not consider its emissions, while for unsustainable biomass we consider its emissions. Sustainable biomass, including bagasse, biogas and biodiesel, is considered green throughout whole life cycle and in line with the perspective of IEA. As such, we don't factor in emissions related to the use of sustainable biomass. Obtaining firewood and charcoal by logging not only degrades forests but also has a detrimental impact on the environment, which is not sustainable, especially in Africa. Thus, we have included this segment of emissions which is similar to the charcoal and primary solid biomass studied by the IEA. It is worth noting that the primary solid biomass we have examined has a narrower scope of coverage compared to the IEA's analysis. For example, we don't consider animal dropping emissions.
